# Glutathione Contribution in Interactions between *Turnip mosaic virus* and *Arabidopsis thaliana* Mutants Lacking Respiratory Burst Oxidase Homologs D and F

**DOI:** 10.3390/ijms24087128

**Published:** 2023-04-12

**Authors:** Katarzyna Otulak-Kozieł, Edmund Kozieł, Krzysztof Treder, Lóránt Király

**Affiliations:** 1 Department of Botany, Institute of Biology, Faculty of Biology and Biotechnology, Warsaw University of Life Sciences—SGGW, Nowoursynowska Street 159, 02-776 Warsaw, Poland; 2 Laboratory of Molecular Diagnostic and Biochemistry, Bonin Research Center, Plant Breeding and Acclimatization Institute—National Research Institute, 76-009 Bonin, Poland; 3 Plant Protection Institute, Centre for Agricultural Research, Eötvös Loránd Research Network (ELKH), 15 Herman Ottó Str., H-1022 Budapest, Hungary

**Keywords:** γ-glutamyl transferase, respiratory burst oxidase homologs, plant–virus interactions, glutathione peroxidase like enzymes, glutathione reductase, glutathione S-transferase

## Abstract

Respiratory burst oxidase homologs (*Rboh*s) play crucial and diverse roles in plant tissue-mediated production of reactive oxygen species during the development, growth, and response of plants to abiotic and biotic stress. Many studies have demonstrated the contribution of *RbohD* and *RbohF* in stress signaling in pathogen response differentially modulating the immune response, but the potential role of the *Rboh*s-mediated response in plant–virus interactions remains unknown. The present study analyzed, for the first time, the metabolism of glutathione in *rbohD-*, *rbohF*-, and *rbohD/F*-transposon-knockout mutants in response to *Turnip mosaic virus* (TuMV) infection. *rbohD–*TuMV and Col-0–TuMV interactions were characterized by susceptible reaction to TuMV, associated with significant activity of GPXLs (glutathione peroxidase-like enzymes) and induction of lipid peroxidation in comparison to mock-inoculated plants, with reduced total cellular and apoplastic glutathione content observed at 7–14 dpi and dynamic induction of apoplast GSSG (oxidized glutathione) at 1–14 dpi. Systemic virus infection resulted in the induction of *AtGSTU1* and *AtGSTU24*, which was highly correlated with significant downregulation of GSTs (glutathione transferases) and cellular and apoplastic GGT (γ-glutamyl transferase) with GR (glutathione reductase) activities. On the contrary, resistant *rbohF–*TuMV reactions, and especially enhanced *rbohD/F–*TuMV reactions, were characterized by a highly dynamic increase in total cellular and apoplastic glutathione content, with induction of relative expression of *AtGGT1*, *AtGSTU13*, and *AtGSTU19* genes. Moreover, virus limitation was highly correlated with the upregulation of GSTs, as well as cellular and apoplastic GGT with GR activities. These findings clearly indicate that glutathione can act as a key signaling factor in not only susceptible *rbohD* reaction but also the resistance reaction presented by *rbohF* and *rbohD/F* mutants during TuMV interaction. Furthermore, by actively reducing the pool of glutathione in the apoplast, GGT and GR enzymes acted as a cell first line in the Arabidopsis–TuMV pathosystem response, protecting the cell from oxidative stress in resistant interactions. These dynamically changed signal transductions involved symplast and apoplast in mediated response to TuMV.

## 1. Introduction

Plant NADPH oxidases (NOXs) are known as respiratory burst oxidases and are encoded by *Rboh* genes [1,2]. Respiratory burst oxidases can transfer electrons to extracellular O_2_ to produce O_2_—which can be converted to H_2_O_2_ by superoxide dismutase [3]. Therefore, *Rboh*s are considered as important factors in the reactive oxygen species (ROS) signaling network. Analysis of the expression of *Rboh*s genes may help to comprehend the possible role of the *Rboh*s family. In Arabidopsis, Sagi et al. [4] observed high expression of *RbohA* to *RbohG* and *RbohI* in roots, *RbohH* and *RbohJ* in pollen and stamen, and *RbohD* and *RbohF* in all plant tissues. *Rboh* was first identified in rice (*Oryza sativa*) and called *OsRbohA* [5]. In general, *Rboh*s are encoded by multiple genes. Thus far, 10 *Rboh* genes have been found in *Arabidopsis thaliana* [4]. Moreover, increasing data suggest that *Rboh*-mediated ROS production is critical for plant development, growth, and response to abiotic and biotic stress [3,6,7,8,9,10,11,12]. *Rboh*s are a major source of ROS during plant–pathogen interactions [13]. Among *Rboh*s, *RbohD* and *RbohF* are pleiotropic and can function together, contributing to stress signaling, especially pathogen response differentially modulating the immune response [14]. Many studies have reported the essential role of *Rboh*-mediated ROS generation in cell death and hypersensitive response (HR) resistance in different fungal [15,16], bacterial [17], or oomycete interactions [18,19]. It has been demonstrated that *Rboh* isoforms performed different roles in the same host or different hosts, but they differentially regulated signaling pathways in plant immune response as a resistance to pathogens [20,21,22].

Although plant–virus interactions were first discovered in a study on oxidative burst in *N. tabacum*–*Tobacco mosaic virus* pathosystem [23], the significance of *Rboh*-mediated response in these interactions remains unclear. In our previous studies, we documented for the first time the induction and subcellular localization of *RbohD* in susceptible response and HR of potato plants to *Potato virus Y* NTN (PVY^NTN^), showing strong accumulation of *RbohD* in HR reaction in apoplast [24]. Our further studies indicated that during development, other Potyvirus, TuMV infection was promoted in *rbohD—A. thaliana* transposon mutant, suggesting that *RbohD* plays a role in Arabidopsis resistance response [25]. Different types of interactions have been observed in *rbohF* and *rbohD/F* double mutants, with a strong limitation of TuMV infection accompanied by a lack of virus cytoplasmic inclusions. These findings suggest that *RbohF* promotes viral infection and induces susceptibility. Moreover, studies have highlighted the role of NOX homologs *RbohD* and *RbohF* in the regulation of TuMV infection in Arabidopsis as well as that of *RbohD*-derived ROS in the resistance response in TuMV–Arabidopsis interactions [25]. Most importantly, beyond transcriptional regulations, studies have shown that the functions of *Rboh*s in plants are regulated or modulated by several signaling molecules [12,26,27,28,29]. Furthermore, with increasing studies on the regulation of *Rboh*s, a higher number of molecules and factors that are regulated and associated with *Rboh*s activity are being identified, but the mechanisms of their regulations remain poorly understood.

Glutathione is an essential metabolite known for its role in the regulation of ROS generation [30]. Free glutathione exists in two reversible states—reduced sulfhydryl form (GSH) and disulfide dimer formed from oxidation (GSSG). Both GSH and GSSG specify the reduced (oxidized) form [31]. Together with ascorbate, glutathione acts as a key component in mechanisms controlling ROS levels in plants [32]. Moreover, some functions of GSH involve reversible redox reactions [33]. When acting as an antioxidant, GSH is oxidized to GSSG, whereas under stress conditions, it is reduced by glutathione reductase (GR) to GSH; therefore, the glutathione pool is mostly reduced. Sabetta et al. [34] underlined that glutathione is a mobile molecule that is systemically transported and can be remobilized when needed. Thus, GSH acts as a mediator of important processes such as cell death in plant cells [35]. Some studies have highlighted the contribution of glutathione in responses to virus inoculation [36,37,38], but data regarding this contribution are scarce. Our previous studies indicated that glutathione acts as a modulator in potato–PVY^NTN^ pathosystem [39]. Moreover, our further studies examined the response of glutathione transferase class *tau* (GSTU)-knockout Arabidopsis mutants *Atgstu19* and *Atgstu24* to TuMV [40]. We assumed that AtGSTU19 and AtGSTU24 are important factors modulating the response of Arabidopsis plants to TuMV. Taken together, our findings suggest that *RbohD* and *RbohF* contribute differently in the reactions to TuMV and different molecules can be associated with the activity of *Rboh*s during pathogen infection. Furthermore, it is clear that glutathione can modulate different plant–virus interactions.

The aim of our study was to verify whether glutathione and glutathione metabolism enzymes play a role in *rbohD–*, *rbohF*–, and *rbohD/F* double mutant–TuMV interactions. The study investigated, for the first time, glutathione localization and content, glutathione-associated relative expression of selected genes, and enzyme activities in *rbohD–*, *rbohF–*, and *rbohD/F*–TuMV pathosystem. The results showed significant differences in cellular and apoplastic glutathione localization and content between susceptible and resistance responses to virus inoculation. Moreover, significant variations were noted in the relative expression of *AtGGT* and *AtGSTUs*, which clearly indicated the role of glutathione and *AtGGT*, *AtGSTUs* in resistance as well as susceptible reaction to TuMV.

## 2. Results

### 2.1. Significant Regulation of TuMV Concentration in Col-0–, rbohD–, rbohF–, and rbohD/F–TuMV Interactions

**Figure 1:**
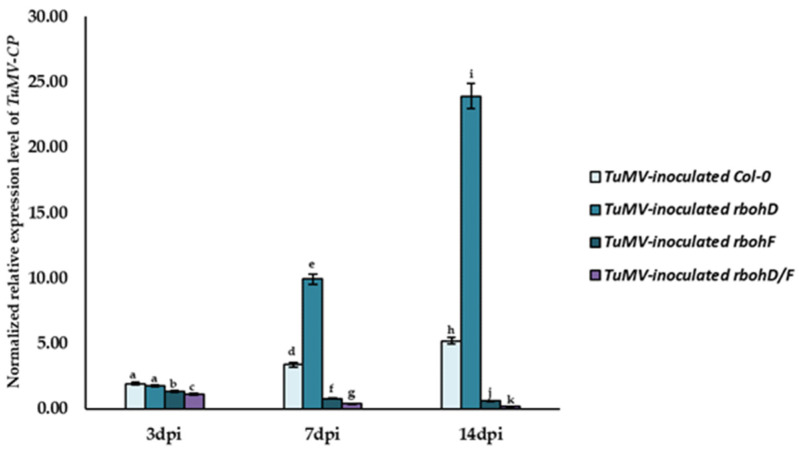
Validation of TuMV levels in Col-0, *rbohD*, *rbohF*, and *rbohD/F* plants at 3, 7, and 14 dpi based on normalized relative expression of *TuMV-CP*. Normalized relative expression of *TuMV-CP* was calculated based on the mean expression of AtEf1α and AtF-Box reference genes. Statistical significance of differences was assessed at the *p* < 0.05 level using ANOVA with post hoc Tukey’s HSD and is indicated by different letters above the bars.

Our previous research demonstrated that Col-0 and *RbohD* and *RbohF* transposon mutants *rbohD*, *rbohF*, and *rbohD/F* exhibited different levels of susceptibility/resistance to TuMV isolate PV-0104 [25]. Moreover, *rbohD* plants displayed systemic virus infection and were even more susceptible than Col-0 plants. On the other hand, *rbohF* plants showed virus limitation and were more resistant to TuMV compared to Col-0. In turn, *rbohD/F* plants displayed even higher resistance than *rbohF* accompanied by local necrosis, which is characteristic of HR-like reactions [25]. Previously, virus titers were checked serologically (DAS-ELISA) and by ultrastructural analysis (TEM). Therefore, in the present study, we performed a validation based on the relative expression of *TuMV-CP* (TuMV capsid protein) gene, in comparison to two plant host reference genes, *AtEf1α* (*A. thaliana* elongation factor-1 alpha) and *AtF-Box* (*A. thaliana* F-box protein family), in order to understand the changes in TuMV content in the inoculated leaves between 3 and 14 dpi (Figure 1). The results of *TuMV-CP* gene expression analysis clearly indicated the upregulation of *TuMV-CP* in Col-0 (2.7-fold) and *rbohD* (13.58-fold) plants between 3 and 14 dpi. On the contrary, in the same time after inoculation, *TuMV-CP* levels were decreased in *rbohF* (2.24-fold) and *rbohD/F* (5.32-fold). This confirms that *rbohD* mutants are more susceptible to TuMV infection than the already susceptible Col-0 plants, while *rbohF* mutants are more resistant to TuMV infection. Moreover, *rbohD/F* plants showed enhanced resistance to TuMV to some extent. The high induction or reduction of virus content was clearly evident at the 7-dpi timepoint in different types of infected plants.

### 2.2. Crucial Cellular and Apoplastic Localization with Concentration of GSH (Reduced Glutathione) and GSSG (Oxidized Glutathione) Pools as an Element of Increased Susceptibility or Resistance Reaction in rbohD, rbohF, and rbohD/F Arabidopsis Mutants

**Figure 2:**
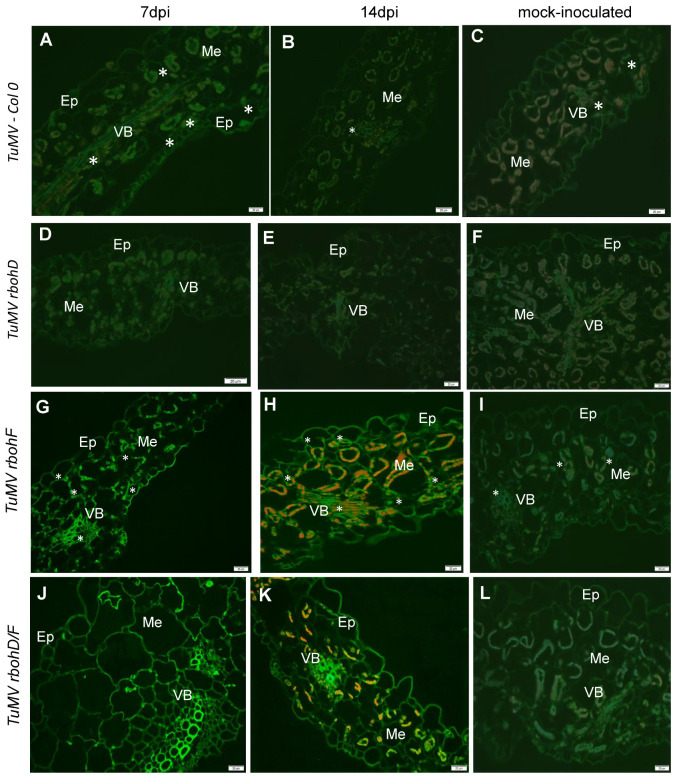
Total glutathione immunofluorescence signals (*) in TuMV-Col-0, -*rbohD*, -*rbohF*, and -*rbohD/F* between 7 (**A**,**D**,**G**,**J**) and 14 dpi (**B**,**E**,**H**,**K**) and mock-inoculated (**C**,**F**,**I**,**L**) leaves. Glutathione signal (*) in mesophyll cells (Me) and vascular bundles (VB) in Col-0 and *rbohD*. In *rbohF* and *rbohD/F* in cell walls of epidermis (Ep) and in protoplast and apoplastic fluids fraction of vascular tissues (VB) and mesophyll cell (Me). (**C**,**H**,**K**) red signal is chlorophyll autofluorescence. Scale bar: 20 μm, Ep—epidermis, Me—mesophyll cells, VB—vascular bundles.

**Figure 3:**
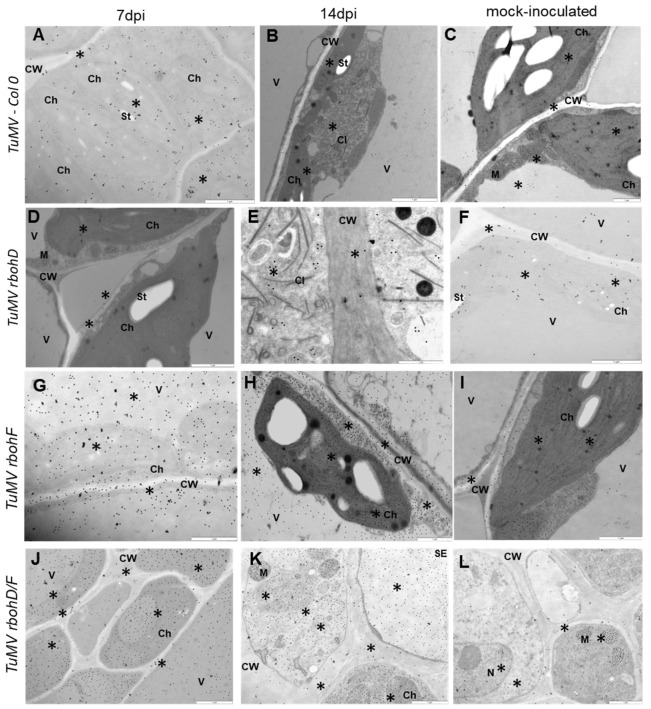
Total glutathione immunogold labeling (*) in TuMV-Col-0, -*rbohD* between 7 (**A**,**D**,**G**,**J**) and 14 dpi (**B**,**E**,**H**,**K**) and mock- inoculated (**C**,**F**,**I**,**L**) leaves. (**A**) Glutathione deposition (*) in cytoplasm and chloroplast of Col-0 spongy mesophyll cells at 7 dpi. Scale bar: 1 μm. (**B**) Glutathione deposition (*) in cytoplasm around TuMV cytoplasmic inclusion (CI) and in chloroplast of palisade mesophyll at 14 dpi. Scale bar: 1 μm. (**C**) Glutathione deposition (*) in mitochondrion (M), cytoplasm and chloroplast (Ch) of palisade mesophyll cells in mock-inoculated Col-0. Scale bar: 1 μm. (**D**) Glutathione deposition (*) near curved lamellas chloroplasts (Ch). Scale bar: 1 μm. (**E**) Glutathione deposition (*) in cell wall and cytoplasm fulfilled with TuMV cytoplasmic inclusion (CI) at 14 dpi. Scale bar: 1 μm. (**F**) Glutathione deposition (*) in chloroplast and vacuole of mock-inoculated *rbohD*. Scale bar: 1 μm. (**G**) Glutathione deposition (*) in cell wall, vacuole, and chloroplasts at 7 dpi. Scale bar: 1 μm. (**H**) Glutathione deposition (*) in cell wall (CW), multivesicular structures, vacuole (V), and chloroplast (Ch) in palisade mesophyll cells at 14 dpi. Scale bar: 1 μm. (**I**) Glutathione deposition (*) in chloroplast (Ch) and cytoplasm near cell wall (CW) of palisade mesophyll from mock-inoculated *rbohF*. Scale bar: 1 μm. (**J**) Glutathione deposition (*) in cell walls (CW) and protoplasts of phloem cells at 7 dpi. Ch- chloroplasts. Scale bar: 1 μm. (**K**) Glutathione deposition (*) in sieve element (SE) and cell walls (CW), mitochondria (M), and chloroplasts (Ch) of phloem parenchyma cells at 14 dpi. Scale bar: 1 μm (**L**) Glutathione deposition (*) in cell walls (CW), mitochondria (M), and nucleus (N) inside phloem cells of mock-inoculated *rbohF/D*. Scale bar: 1 μm.

**Figure 4:**
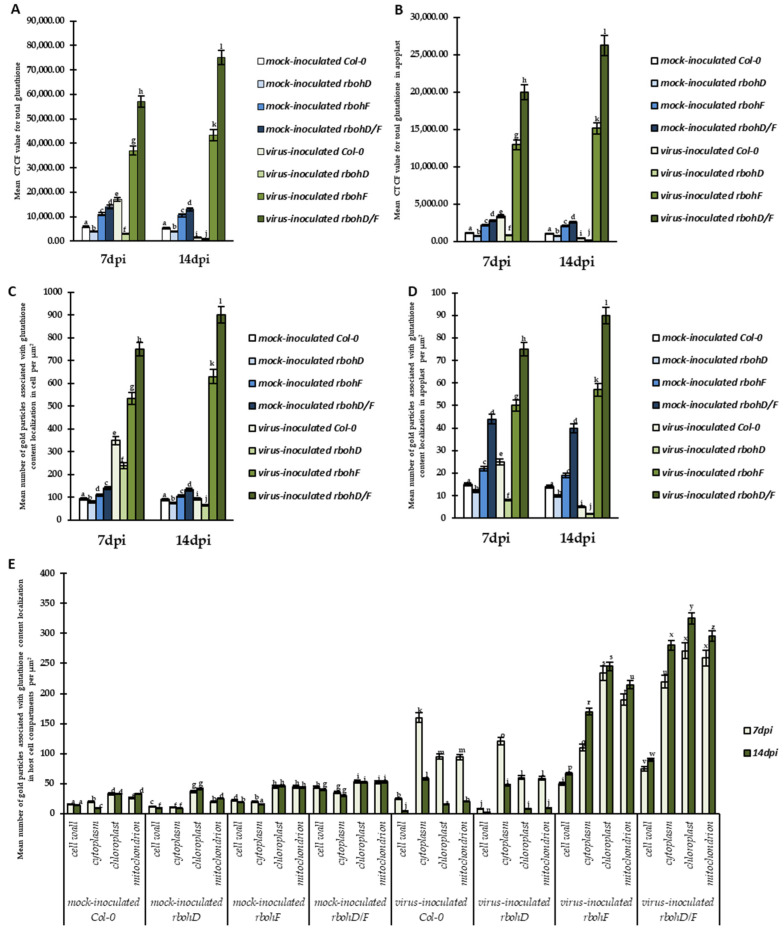
Quantification of total glutathione with the use of: fluorescence signals based on cell total corrected fluorescence and quantified immunogold labeling in the cell (**A**,**C**), apoplast (**B**,**D**) and cell compartments (**E**) in TuMV- and mock-inoculated Col-0, *rbohD*, *rbohF*, and *rbohD/F* plants between 7 and 14 dpi. Using ANOVA and Tukey’s HSD test, the mean fluorescence/mean number of gold particles µm^2^ of GSH and GSSG was calculated at *p* < 0.05. Statistically significant values are indicated by different letters above the bars.

**Figure 5:**
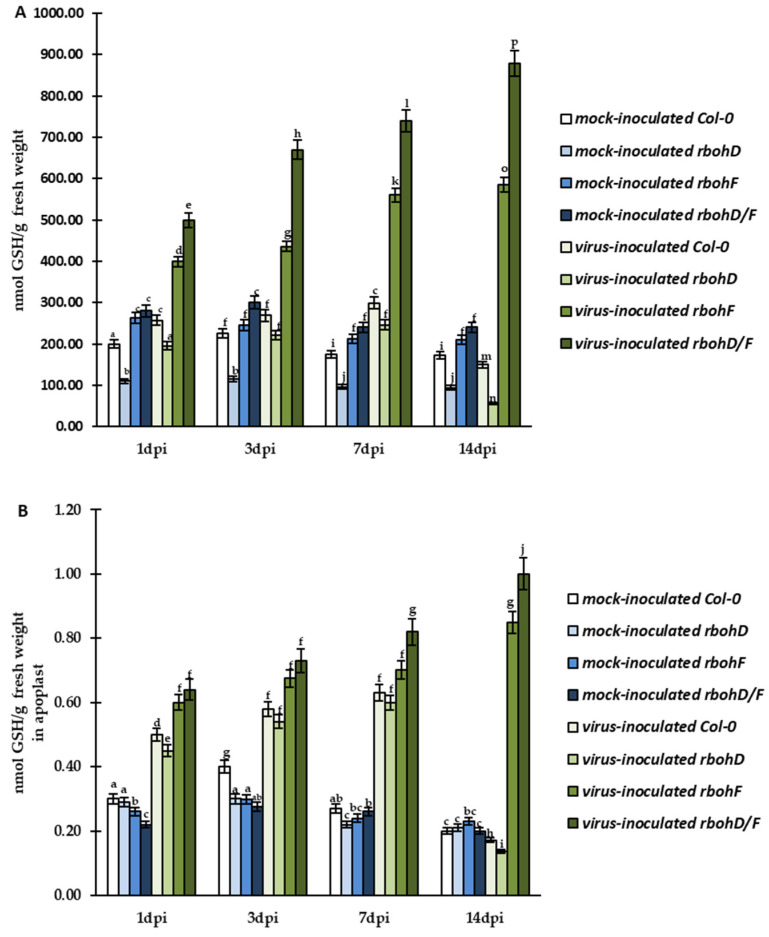
The mean concentration of cellular GSH (**A**) and apoplastic GSH (**B**) in the leaves of TuMV- and mock-inoculated Col-0, *rbohD*, *rbohF*, and *rbohD/F* plants between 1 and 14 dpi. Using ANOVA and Tukey’s HSD test, the mean concentrations of GSH and GSSG were calculated at *p* < 0.05. Statistically significant values are indicated by different letters above the bars.

**Figure 6:**
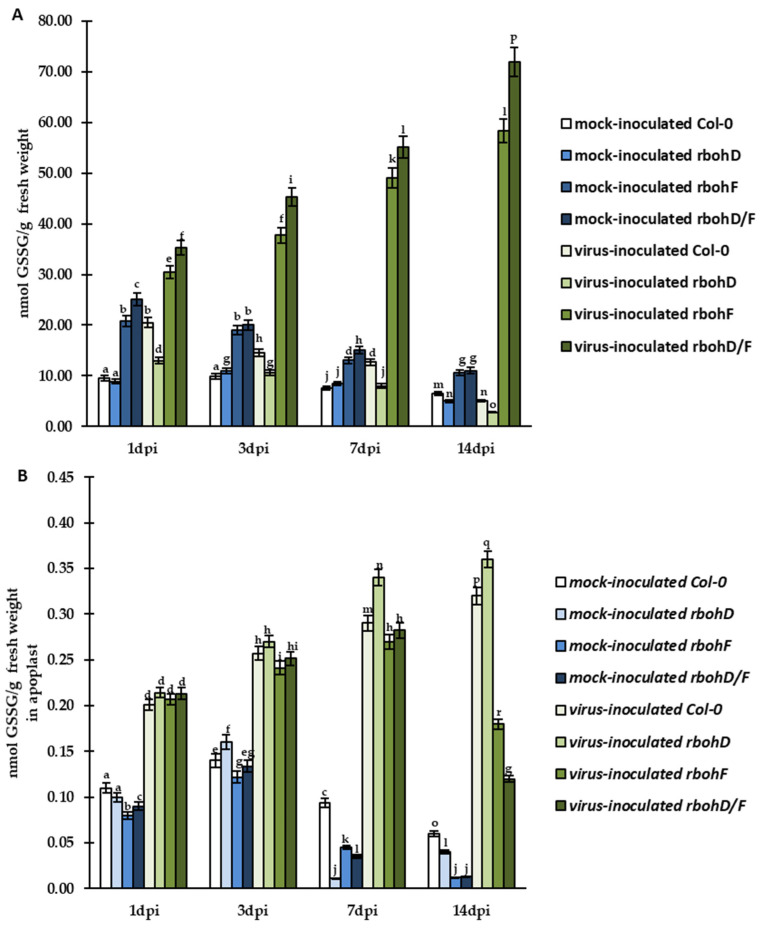
The mean concentration of GSSG in cell (**A**) and apoplast (**B**) in the leaves of TuMV- and mock-inoculated Col-0, *rbohD*, *rbohF*, and *rbohD/F* plants between 1 and 14 dpi. Using ANOVA and Tukey’s HSD test, the mean concentrations of GSH and GSSG were calculated at *p* < 0.05. Statistically significant values are indicated by different letters above the bars.

**Figure 7:**
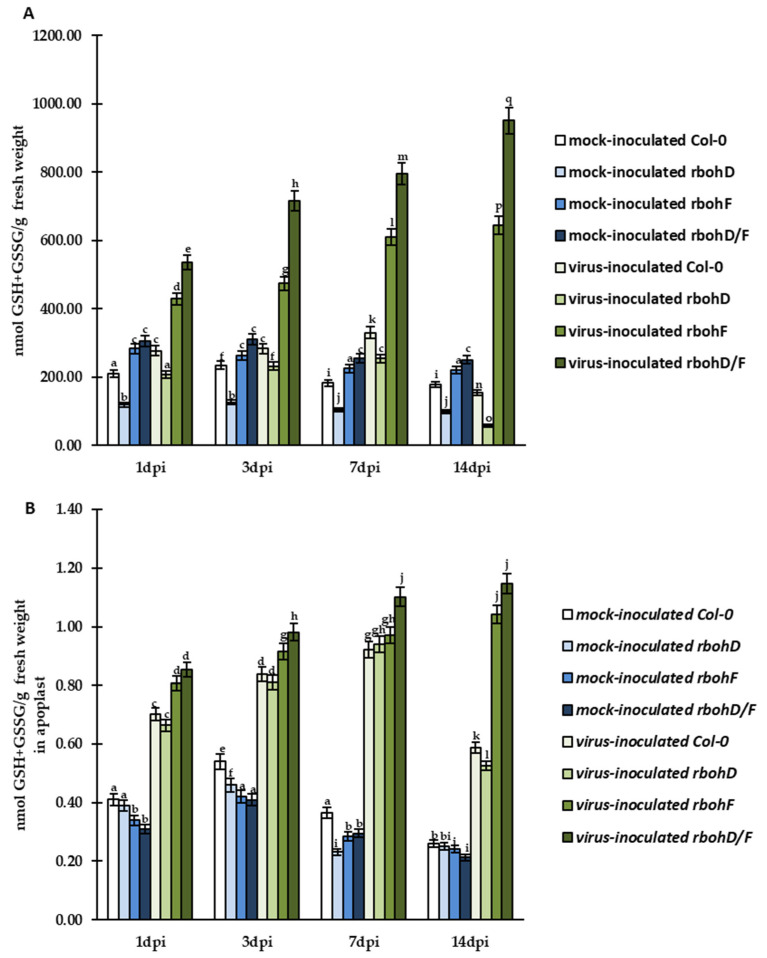
The mean total concentration of GSH and GSSG (GSH+GSSG) in cells (**A**) and apoplast (**B**) in the leaves of TuMV- and mock-inoculated Col-0, *rbohD*, *rbohF*, and *rbohD/F* plants between 1 and 14 dpi. Using ANOVA and Tukey’s HSD test, the mean concentrations of GSH and GSSG were calculated at *p* < 0.05. Statistically significant values are indicated by different letters above the bars.

The changes in the expression of *TuMV-CP* during TuMV infection in *rbohD*, *rbohF*, and *rbohD/F* mutant plants confirmed the differences in the reaction of these plants to the presence of TuMV. Moreover, our previous works confirmed that *RbohD* [24] and glutathione [39,40] act as modulators of reaction to different Potyviruses. In normal physiological conditions, the levels of *Rboh*s and glutathione are precisely regulated in plants. Furthermore, the changes in their levels are highly correlated. This leads to the question on how glutathione levels will be changed and whether glutathione levels will be correlated with the modulation of host reactions, namely susceptibility or resistance, to TuMV infection. To answer this question, we quantified immunofluorescence and immunogold localization of total glutathione content (Figure 2 and Figure 3, respectively) with the use of corrected total cell fluorescence (CTCF) estimation (Figure 4A,B) and calculation of mean number of gold particles (Figure 4C,D). As an expansion of these estimations, HPLC (high-performance liquid chromatography) analyses were performed, to calculate the cellular and apoplastic (Figure 5A,B, Figure 6A,B and Figure 7A,B) levels of GSH, GSSG pools during ongoing TuMV infection in Col-0 and all types of mutant plants. Immunofluorescence localization and validation of total glutathione fluorescence signal by CTCF showed different localization of total glutathione in the cell and apoplast (Figure 2A–L and Figure 4A,B) between 7 and 14 dpi, indicating that TuMV inoculation induced GSH deposition in Col-0 and mutant plants compared to mock-inoculated plants (Figure 2A–L and Figure 4A–D). Moreover, total glutathione was mainly observed in the protoplast of parenchymal and vascular bundle cells in Col-0 and *rbohD* mutant plants (Figure 2A–F), whereas it was observed in all leaf tissues in *rbohF* and *rbohD/F* mutants (Figure 2G–L). The strength of the fluorescence signal of total glutathione was also significantly higher in symplast as well as in apoplast of TuMV-inoculated *rbohF* and *rbohD/F* plants (Figure 2G–L and Figure 4A,B), with the strongest signal observed in the cell wall of vascular bundles. In contrast, in virus-inoculated Col-0 and *rbohD* plants, a dynamic and statistically significantly decreased strength of fluorescence signal was observed from 7 to 14 dpi in cell and in the apoplast region. Validation of total glutathione fluorescence signal by CTCF showed the regulation of total cellular and apoplastic glutathione (Figure 4A,B) between 7 and 14 dpi.

The total glutathione immunogold localization (Figure 3A–L) confirmed CTCF analyses data. TuMV-inoculated Col-0 and *rbohD* plants were characterized by a significantly decrease of glutathione in symplast as well as in apoplast between 7 and 14 dpi (Figure 3A,B,D,E and Figure 4C–E). Moreover, TuMV-inoculated Col-0 and *rbohD* plants characterized also decreased deposition in specific cell compartments (mitochondria and chloroplast) between 7 and 14 dpi. Whereas, deposition of immunogold particles associated with glutathione was statistically increased in symplast as well as in apoplast of TuMV-inoculated *rbohF* and *rbohD/F* plants was induced between 7 and 14 dpi (Figure 3G,H,J,K and Figure 4C,D). In contrary to the Col-0 and *rbohD* plants, TuMV-inoculated *rbohF* and *rbohD/F* plants had also induced deposition in cytoplasm, mitochondria, and especially chloroplast between 7 and 14 dpi (Figure 4E).

Further quantitative HPLC analyses confirmed significant and timepoint-specific regulation of both glutathione (GSH and GSSG) forms in cells and in the apoplast of Col-0, *rbohD*, *rbohF*, and *rbohD/F* during TuMV infection. During virus infection, we observed enhanced GSH content in cell and apoplast from 1 to 7 dpi in comparison to mock-inoculated Col-0 and mutant plants (Figure 5A,B). However, this increase was more dynamic and significant in *rbohF*–TuMV (resistance) and *rbohD/F*–TuMV (enhanced resistance) pathosystems. The highest levels of GSH were always found in virus-inoculated *rbohD/F* plants (Figure 5A,B). Moreover, GSH was continuously induced between 1 and 14 dpi in *rbohF* (1.47-fold in cell, 1.42-fold in apoplast) and *rbohD/F* (1.76-fold in cell, 1.56-fold in apoplast) plants. Unlike resistant *rbohF* and *rbohD/F* plants, in the virus-inoculated Col-0 (susceptibility) and *rbohD* (enhanced susceptibility) plants, enhanced cellular and apoplastic GSH content was observed only up to 7 dpi, after which the GSH level decreased (Figure 5A,B). Most interestingly, in virus-inoculated *rbohD* plants, the decrease of GSH (4.45-fold in cell, 4.37-fold in apoplast) was more evident and intense than that observed in TuMV-susceptible Col-0 plants. Furthermore, the regulation of cellular and apoplastic GSH levels in these two susceptible plants was highly associated with the 7-dpi time point.

GSH content as well as the cellular and apoplastic levels of GSSG was differentially regulated during TuMV infection (Figure 6A,B). In resistant TuMV-inoculated *rbohF* and *rbohD/F* plants, the cellular GSSG content was systemically induced between 1 and 14 dpi by 1.92-fold and 2.04-fold, respectively, compared to mock-inoculated plants. However, in the same TuMV-inoculated plants, the apoplast GSSG content was increased only between 1 and 7 dpi (Figure 6B), and after 7 dpi, the GSSG content was decreased by 1.50-fold and 2.35-fold, respectively. This indicates that TuMV inoculation modulated GSH content, which was probably due to direct conversion of GSH to GSSG in cells, but in the apoplast, another factor was also involved in GSSG regulation at some point. On the other hand, in virus-inoculated susceptible (Col-0) plants and plants with enhanced susceptibility (*rbohD*), the cellular GSSG level was decreased by approximately 4-fold and 4.71-fold, respectively. In turn, the apoplast level of GSSG was systemically increased between 1 and 14 dpi by 1.59-fold and 1.68-fold, respectively. This suggests that virus-inoculated *rbohD* plants developed more severe reactions in response to the oxidative stress in cell, which may be related to increased levels of GSSG being directed to the apoplast region.

The analyses of CTCF, quantification of immunogold labeling, a and further evaluation of HPLC results revealed that TuMV infection modulated changes in summary glutathione (GSH+GSSG) levels in cell and apoplast (Figure 7A,B). In virus-inoculated *rbohF* and *rbohD/F* plants, there was, respectively, a 1.5-fold (in cells) and 1.29-fold (in apoplast) and 1.78-fold (in cells) and 1.34-fold (in apoplast) increase of GSH+GSSG pool between 1 and 14 dpi. In contrast, in Col-0- and *rbohD*-TuMV plants, cellular and apoplastic GSH+GSSG levels dynamically decreased between 7 and 14 dpi, with the highest decrease in *rbohD* (4.37-fold in cells and 1.78-fold in apoplasts).

The modulation of GSH, GSSG pools, and total GSH+GSSG indicated that current oxidation of plant cells infected by TuMV could be also changed. The most important parameter which is associated with oxidation feature of cell is GSH/GSSG ratio. The TuMV inoculated Col-0 and *rbohD* were characterized by slight increase in GSH/GSSG ratio between 1 and 7 dpi (Figure S1). Conversely, the GSH/GSSG ratio was drastically reduced at 7 dpi to the lowest levels in the case of *rbohD* plants. In contrast to that, TuMV-infected *rbohF* and *rbohD/F* plants were characterized by a systematic increase of the GSH/GSSG ratio between 1 and 14 dpi.

### 2.3. Relative Expression of AtGGT1 and Selected AtGSTU Genes during Col-0–, rbohD–, rbohF–, and rbohD/F–TuMV Interactions Correlated with Increased Susceptibility or Resistance Reaction

The results from CTCF quantification of immunolocalization of total glutathione indicated decreased deposition of glutathione near the apoplasts of infected cells in Col-0 and *rbohD* plants and increased deposition in *rbohF* and *rbohD/F* plants inoculated with virus. This suggests that modulation of cellular and apoplastic glutathione could have contributed to the reaction to TuMV infection. The main enzyme responsible for the catabolism/usage of glutathione in cell wall in *A. thaliana* is GGT (γ-glutamyl transferase) [41]. One isoform of this enzyme plays a major role in leaf cell apoplast and is encoded by *AtGGT1* gene [41]. Therefore, we examined the normalized expression of *AtGGT1* gene in cells in the *Atrboh*–TuMV pathosystem. The changes in the expression of this gene could explain the modulation of glutathione content in the cell wall. Analyses of *AtGGT1* relative expression indicated significant changes in TuMV-inoculated plants (Figure 7A). The gene was highly upregulated in virus-inoculated *rbohF* (1.41-fold) and *rbohD/F* (2.25-fold) plants between 3 and 14 dpi, and its expression was also much higher compared to mock-inoculated plants. Moreover, the rate of upregulation and the final level of *AtGGT1* expression was the highest in *rbohD/F* plants. In contrast, virus inoculation induced *AtGGT1* in Col-0 and *rbohD* plants only at 3 dpi compared to mock-inoculated plants. Moreover, the relative expression of *AtGGT1* decreased systemically after 3 dpi by 4.45-fold in virus-inoculated Col-0 and by 21-fold in virus-inoculated *rbohD* plants. Our previous investigation showed that GSTs (glutathione S-transferases), especially class tau (GSTU), play an important role in the modulation of cell glutathione levels in TuMV infection [40]. Moreover, modification of the expression of different GST genes was found to be crucial for the precise modulation of the host’s response to this viral pathogen [40]. Therefore, in this study, we analyzed the relative expression of four GST genes—*AtGSTU1*, *AtGSTU13*, *AtGSTU19*, and *AtGSTU24*—which were previously identified to be involved in response to TuMV infection.

**Figure 8:**
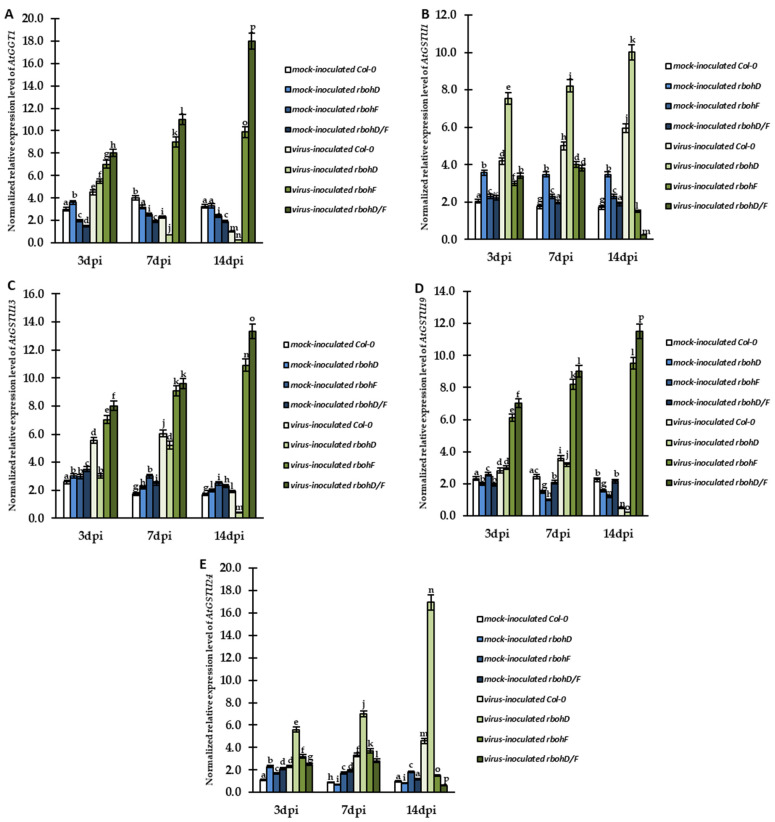
The normalized relative expression levels of *AtGGT1* (**A**), *AtGSTU1* (**B**), *AtGSTU13* (**C**), *AtGSTU19* (**D**), and *AtGSTU24* (**E**) calculated based on the mean expression of *AtEf1α* and *AtF-Box* reference genes in mock- and virus-inoculated Col-0, *rbohD*, *rbohF*, and *rbohD/F* plants between 3 and 14 dpi. The mean values of the normalized expression levels were calculated and analyzed using ANOVA and Tukey’s HSD test at *p* < 0.05. Statistically significant values are indicated by different letters above the bars.

In contrast to *AtGGT1*, the expression of *AtGSTU1* gene was induced between 3 and 14 dpi in TuMV-inoculated Col-0 and *rbohD* plants compared to mock-inoculated plant. The induction was found to be 1.42-fold in virus-inoculated Col-0 and 1.33-fold in virus-inoculated *rbohD* plants (Figure 8B). The final expression level at the 14-dpi timepoint was the highest in TuMV-inoculated *rbohD* plants. On the contrary, in *rbohF*–TuMV and *rbohD/F*–TuMV interactions, *AtGSTU1* expression was upregulated only between 3 and 7 dpi (in comparison to mock-inoculated plants). After 7 dpi, the level of *AtGSTU1* decreased by 2.67-fold and 16-fold, respectively, in virus-inoculated *rbohF* and *rbohD/F* plants. The relative expression of *AtGSTU13* was also significantly modulated between 3 and 14 dpi in Col-0, *rbohD*, *rbohF*, and *rbohD/F* plants after TuMV inoculation (Figure 8C). In the *rbohF*– and *rbohD/F*–TuMV interaction, we observed induction of *AtGSTU13* expression compared to mock-inoculated plants, and the induction was the highest in *rbohD/F* plants (Figure 8C). In contrast, the expression of *AtGSTU13* was induced in virus-inoculated Col-0 and *rbohD* only between 3 and 7 dpi, and after 7 dpi, it significantly reduced. Further analyses of the relative expression of *AtGSTU19* and *AtGSTU24* (Figure 8D,E) showed significant changes in the analyzed plants. The timepoint changes in *AtGSTU19* expression were similar to the modulation of *AtGSTU13* expression. The normalized expression of *AtGSTU19* was highly upregulated in *rbohF* and *rbohD/F* plants during interaction with TuMV by 1.55-fold and 1.64-fold, respectively, between 3 and 14 dpi. On the other hand, in Col-0 and *rbohD* plants, the expression of *AtGSTU19* was downregulated by 7.24-fold (Col-0) and 13.85-fold (*rbohD*) after 7 dpi. The time of regulation of *AtGSTU24* was similar to that of *AtGSTU1. AtGSTU24* was upregulated between 3 and 14 dpi in Col-0 (2-fold) and *rbohD* (3.03-fold) plants during interaction with TuMV (Figure 8D), whereas its expression was only slightly induced between 3 and 7 dpi in *rbohF* and *rbohD/F* plants during TuMV interaction. After 7 dpi, *AtGSTU24* was downregulated in virus-infected *rbohF* and *rbohD/F* plants.

### 2.4. Changes in GGT and GST, with GR and GPXL Activity, as Modulators of Enhanced Susceptibility and Resistance during rbohD–, rbohF–, and rbohD/F–TuMV Interactions, along with Control of Lipid Peroxidation

**Figure 9:**
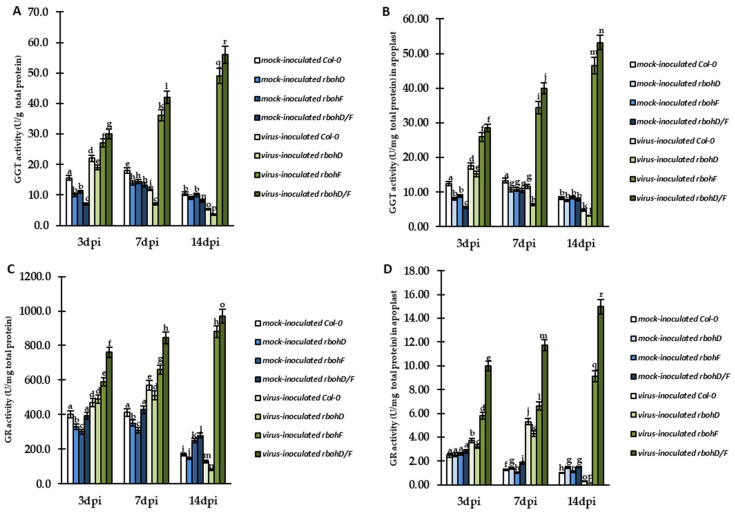
Cellular and apoplastic activity of GGT (**A**,**B**) and GR (**C**,**D**) in the leaves of TuMV- and mock-inoculated Col-0, *rbohD*, *rbohF*, and *rbohD/F* plants between 3 and 14 dpi. The mean activities (in U/mg total protein) were calculated and analyzed by ANOVA and Tukey’s HSD test at *p* < 0.05. Statistically significant values are indicated by different letters above the bars.

The modulation of total glutathione localization, with changed levels of cellular and apoplastic GSH/GSSG forms, and differences in the expression of *AtGTT1* and *AtGSTU* genes, revealed the precise control of glutathione in different types of host reactions to TuMV. Therefore, we analyzed the cellular and/or apoplastic activity of glutathione metabolism enzymes in reaction to viral infection. For validation, we selected enzymes associated with whole cell (GSTU, GR, and glutathione peroxidase-like enzymes (GPXLs)) as well as two enzymes associated mainly or partially with the apoplast of host cells (GGT and GR). The analyses of the activity of GGT (Figure 9A,B) and GR (Figure 9C,D) indicated other types of relationship with different reactions to TuMV. The activity of cellular GGT was stably upregulated between 3 and 14 dpi in virus-inoculated *rbohF* and *rbohD/F* mutants, respectively, by 1.81-fold and 1.87-fold (Figure 9A). Similarly, upregulated GGT activity was also observed in apoplasts in *rbohF* and *rbohD/F* mutants (Figure 9B) In these plants, GGT activity was induced, respectively, by 1.8-fold and 1.87-fold. In contrast, in virus-inoculated Col-0 and *rbohD* plants, cellular GGT activity was induced only at 3 dpi, compared to mock-inoculated plants. After 3 dpi, GGT activity was found to be decreased in these plants by 4.17-fold (Col-0) and 5.43-fold (*rbohD*). The same pattern was also observed for the activity of GTT in apoplast, with depletion of enzyme activity by 3.71-fold (Col-0) and 4.83-fold (*rbohD*). In Col-0 (susceptibility) and *rbohD* (enhanced susceptibility) plants, GR activity was upregulated between 3 and 7 dpi during interaction with TuMV (Figure 9C,D). On the other hand, the upregulation of GR activity in the virus-inoculated *rbohD* plants was lower compared to Col-0 plants and the enzyme activity was only induced by 1.04-fold between 3 and 7 dpi. After 7 dpi, the cellular and apoplastic GR activity was downregulated in Col-0 as well as *rbohD* plants. Moreover, the most significant reduction in cellular (9.72-fold) and apoplastic GR activity (28-fold) was observed in *rbohD* plants. In contrast, *rbohF* and *rbohD/F* plants were characterized by increased cellular and apoplastic GR activity between 3 and 14 dpi (Figure 9C,D). Moreover, the highest induction of enzymatic activity was observed in *rbohD/F* plants during TuMV interaction, with 1.28-fold and 1.5-fold induction in cell and apoplast, respectively.

**Figure 10:**
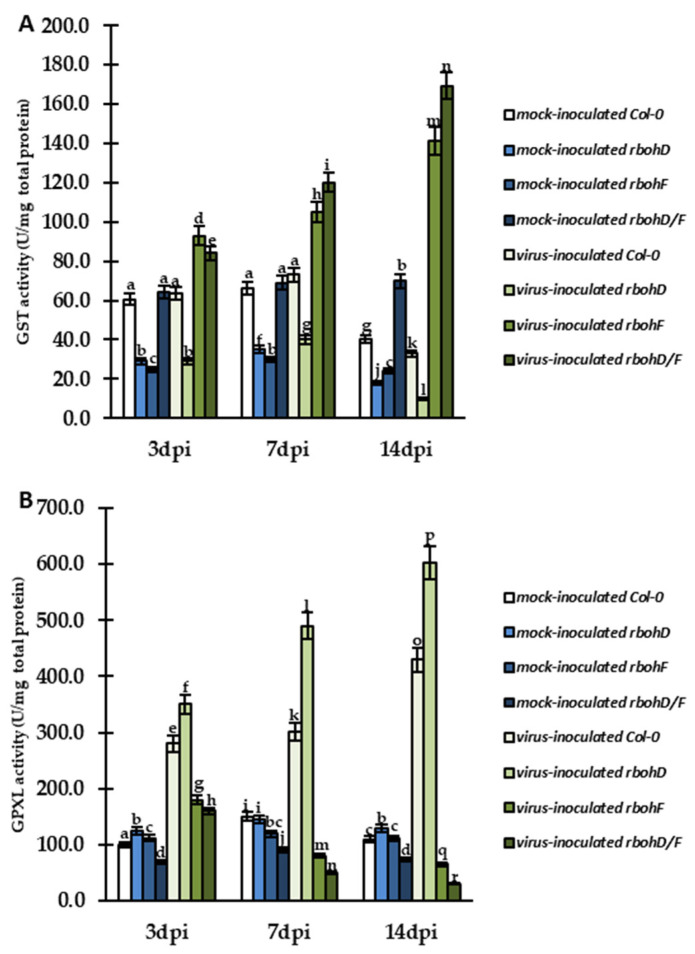
Activity of GST (**A**) and GPXL (**B**) in the leaves of TuMV- and mock-inoculated Col-0, *rbohD*, *rbohF*, and *rbohD/F* plants between 3 and 14 dpi. The mean activities (in U/mg total protein) were calculated and analyzed by ANOVA and Tukey’s HSD test at *p* < 0.05. Statistically significant values are indicated by different letters above the bars.

Further validation of GST (Figure 10A) and GPXL (Figure 10B) activity was also performed. In general, the pattern of GST activity changes was quite similar to that of GR in cell. Plants showing susceptibility (Col-0)/enhanced susceptibility (*rbohD*) during interaction with TuMV had slightly upregulated GST between 3 and 7 dpi (Figure 10A). In the virus-inoculated *rbohD* plants, the activity of GST was lower than in Col-0 (Figure 10A). After 7 dpi, GST activity was downregulated in Col-0 and *rbohD* plants, especially in the latter, in which reduction was 4-fold. In contrast, plants showing resistance (*rbohF*) and enhanced resistance (*rbohD/F*) were characterized by increased activity of GST between 3 and 14 dpi (Figure 10A). The induction of enzymatic activity was the highest (2.12-fold) in *rbohD/F* plants during TuMV interaction. GPXL was upregulated in Col-0 and *rbohD* plants inoculated with TuMV between 3 and 14 dpi, by 1.55-fold and 1.72-fold, respectively (Figure 10B). In turn, GPXL activity was downregulated between 3 and 14 dpi in *rbohF*– and *rbohD/F*–TuMV interaction by 2.77-fold and 5.33-fold, respectively.

**Figure 11:**
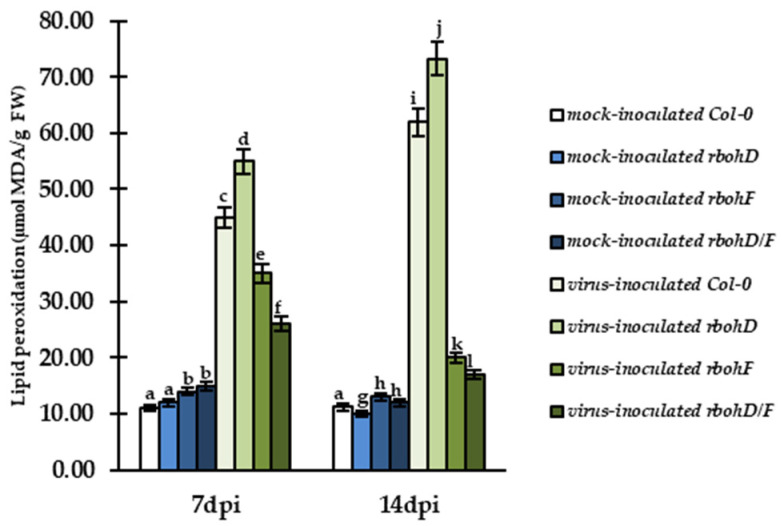
Validation of lipid peroxidation based on the amount of MDA in the leaves of TuMV- and mock-inoculated Col-0, *rbohD*, *rbohF*, and *rbohD/F* plants between 7 and 14 dpi. The mean amount of MDA (µmol MDA/g FW) was calculated and analyzed by ANOVA and Tukey’s HSD test at *p* < 0.05. Statistically significant values are indicated by letters above the bars.

Changes in the cellular and apoplastic activity of glutathione metabolism enzymes suggested that they perform a potential protecting function against oxidative stress and lipid peroxidation in infected plants. To verify this hypothesis, we validated lipid peroxidation based on the amount of malondialdehyde (MDA; Figure 11), as described by Hoges et al. [42]. For this analysis, we selected two time points—7 and 14 dpi—which were highly associated with changes in virus levels during infection.

This validation showed increased levels of MDA (increased lipid peroxidation) in all types of virus-inoculated plants in comparison to mock-inoculated plants at 7 and 14 dpi. However, in virus-inoculated Col-0 (susceptibility) and *rbohD* (increased susceptibility), the levels of MDA were systemically increased by, respectively, 1.38-fold and 1.33-fold at these timepoints, with the highest increase observed in TuMV-inoculated *rbohD* plants. In contrast, MDA levels were decreased between 7 and 14 dpi in TuMV-inoculated *rbohF* (1.75-fold) and *rbohD/F* (1.53-fold) plants and the levels were slightly higher than in mock-inoculated plants. The presented results of cellular and apoplastic activity of glutathione-associated enzymes and lipid peroxidation indicate that precise control of glutathione in both cell regions is crucial for the modulation of host reaction to TuMV infection. Furthermore, it appears that increased levels of GSH and cellular and apoplastic activity of GGT and GR could be responsible for lower lipid peroxidation in plants exhibiting resistance, such as *rbohF* and *rbohD/F*, during ongoing TuMV infection.

To summarize and confirm the relationship between the response of Arabidopsis *RbohD*- and *RbohF*-deficient mutant to TuMV infection and glutathione metabolism-associated findings, a correlation analysis (based on Pearson’s correlation coefficients—PCCs) was performed (Tables S1–S3). PCCs confirmed a high positive correlation between the relative expression of *AtGSTU1* and *AtGSTU24* genes (Table S1) with GPXL activity (Table S2) and the TuMV concentration in Col-0 and *rbohD* plants between 7 and 14 dpi. For the same plants, PCCs indicated a high negative correlation between virus concentration and cellular/apoplastic total glutathione localization and content (Tables S1 and S3), associated with changes in the relative expression of *AtGGT1*, *AtGSTU13*, and *AtGSTU19* genes (Table S1), followed by apoplastic GGT and GR activity with cellular GGT, GSTU, and GR activity (Tables S2 and S3). Unlike *rbohF*– and *rbohD/F*–TuMV interactions, where the virus limitation had a high negative correlation with the relative expression of *AtGSTU1* and *AtGSTU24* genes (Table S1) and the activity of GPXL (Table S2), a high positive correlation of all other glutathione-associated parameters was observed at 7 and 14 dpi (Tables S1–S3). Moreover, PCCs data indicated the importance of glutathione content with the expression of glutathione-associated genes and the activity of selected glutathione metabolism enzymes in cellular as well as apoplastic element of resistance and enhanced resistance reaction in *A. thaliana rbohF*– and *rbohD/F*–TuMV pathosystems.

## 3. Discussion

We analyzed the correlation between the response of *rbohD* and *rbohF Arabidopsis thaliana* mutants to TuMV and glutathione metabolism. *RbohD* and *RbohF* displayed differential contribution to *A. thaliana* reaction to virus inoculation. The present study confirmed reduced relative expression of TuMV during TuMV–*rbohD/F* and TuMV–*rbohF* interactions. In contrast, we observed that *rbohD* supported TuMV infection even more than Col-0, which led to systemic development of virus infection. Therefore, we postulated that *rbohD* mutants are characterized by opposite effects in *A. thaliana*–TuMV interactions in comparison to the and *rbohF* and *rbohD/F*. Moreover, when mutants with *RbohD* defect reacted as more susceptible, *RbohD* appears to play an important role in the resistance response to potyviruses such as TuMV and PVY [24,25]. However, *Rboh* can have different contributions in different pathosystems [43]. In accordance with our results, Lukan et al. [44] indicated that *RbohD* ortholog limited the systemic transport of PVY^N^-GFP to uninoculated potato leaves. In contrast, *RbohD* did not show play any role as a resistance factor in *rbohD*–*Alternaria brassicola* interactions [45]. Moreover, the response of *rbohD* and *rbohF* mutants was not associated with bacteria or necrotrophic fungi promoting spread [15,46]. On the other hand, we demonstrated that *rbohF* and *rbohD/F* mutant exhibited strong TuMV limitation. It can be assumed that *RbohF* promotes virus infection and induces susceptible response to TuMV in Arabidopsis. A similar association between *rboh* mutants and activated resistance was reported in comparison to the wild type [46,47], but often in the context of immunity establishment. This dominant effect of *rbohF* mutation clearly indicates that *RbohF* can act as a susceptibility factor in TuMV infection. The functions of *Rboh*s as well as the mechanism of the signaling modulation process have been analyzed in studies on plant responses to pathogens, but this topic still seems to be poorly understood in terms of plant–virus interactions. The glutathione induction was found to be much more dynamic in HR than in mock-inoculated plants and compared to susceptible potato response and was accompanied by statistically significant GSH induction and deposition in cell wall in HR reaction [39]. Moreover, the response of *Atgstu19*- and *Atgstu24*-knockout mutants to TuMV demonstrated that glutathione and glutathione metabolism enzymes play an important role and can be differentially modulated in the plant–virus pathosystem [40]. A quite similar tendency was reported by Hakmaoui et al. [48], who observed that during *Nicotiana benthamiana*–*Pepper mild mottle virus* interactions the GSH content decreased and systemic infection was suppressed. This finding is also in line with that of Hernandez [49], who highlighted that glutathione was downregulated to efficiently detoxify ROS in a susceptible reaction to prevent the development of symptoms. Therefore, in our experiment, we examined glutathione content and immunolocalization in Col-0, *RbohD*-deficient mutant during susceptible TuMV interactions. Analyses of fluorescence signal and immunogold labeling indicating the role of glutathione in *rbohD*–TuMV and Col-0–TuMV interactions clearly showed that GSH was induced only up to 7 dpi and was located mainly in symplast (usually in cytoplasm). Conversely, between 7 and 14 dpi, its content was significantly decreased. Cellular GSH pool more intensely increased between 1 and 7 dpi in Col-0–TuMV interaction and then in *rbohD*–TuMV interaction compared to mock-inoculated plants and a dynamic decrease was seen between 7 and 14 dpi in cells as well as in apoplasts. On the other hand, GSSG pool content in apoplast steady increased (1–14 dpi) in *rbohD*–TuMV and Col-0–TuMV interactions, but a steady decrease in total cell pool was evident in both susceptible interactions. This reaction reflected also in GSH/GSSG ratio which decreased after 7 dpi indicating the high level of oxidative stress. Decrease of GSSG in *rbohD*–TuMV as well as in Col-0–TuMV interaction was similar to tendency observed in *Atgstu19*–TuMV interactions. It can be assumed that some part of glutathione pool in apoplast constitutes GSSG during susceptible *rbohD*–TuMV and Col-0–TuMV interactions. Therefore, we postulated that the susceptible reaction of Col-0 and especially *rbohD* to TuMV was correlated with the upregulation of TuMV expression, decrease of whole glutathione content (7–14 dpi), and cellular GSSG between 1 and 14 dpi, in contrast to the induction of GSSG, but only in apoplasts. Our observation was similar to the findings from analyses from other pathosystems, in which GSH or/and GSSG pool decrease in mutant or nonmutant plants caused susceptibility reaction to *Pseudomonas* [50,51,52,53].

A completely different situation was observed for the resistance reaction during *rbohF*–TuMV and *rbohD/F*–TuMV interactions. Both interactions were characterized by a highly dynamic increase in glutathione localization (7–14 dpi) in mitochondria and chloroplast, as well as a glutathione content between 1 and 14 dpi after TuMV infection. A similar upregulation of the whole glutathione content was observed during potato resistance reaction to PVY^NTN^ [39] as well as in *Atgstu24*-knockout mutant–TuMV interactions. Moreover, reduced glutathione pool was significantly induced in whole cells and in apoplast. In turn, despite the upregulation of cellular GSSG, in apoplasts, the oxidized glutathione pool increased only to 7 dpi. Furthermore, even based only on fluorescence signal or immunogold labeling, it seems clear that in resistance reaction of *rbohF* and *rbohD/F* to TuMV a strong glutathione localization was associated with whole cell of all Arabidopsis leaf tissues, as well as with apoplast. Our previous ultrastructural observations indicated that *Atgstu24*–TuMV interaction as resistance reaction was characterized by steady stable significant glutathione deposition in cell walls, which was in line with the findings for *rbohD/F*– and *rbohF*–TuMV interactions. Moreover, this statement is consistent with those of Vanacker et al. [54] and Tolin et al. [55], who indicated that in apoplast glutathione acts as a sensor signaling stress conditions, and if the apoplast content is more oxidized, glutathione can take part in adaptation to biotic stress. Our findings from two *Potyvirus* pathosystems are consistent with the reports of Singh et al. [56], who postulated that the GSSG content was higher in resistant plants compared to susceptible ones. Moreover, Kunstler et al. [57] and Kiraly et al. [58] suggested that a high GSSG concentration can indicate the role of glutathione in the suppression of defense response in oxidative stress during resistance reaction to TMV. As stated by Han et al. [59], stress conditions often change the concentration of glutathione as well as causing its shift toward the GSSG form. Therefore, it can be assumed that resistant *rbohF*– and *rbohD/F*–TuMV interactions are characterized by intensively reduced virus expression which is strictly correlated with strong induction of total glutathione pool and GSH/GSSG ratio.

Significant differences in the expression profile of selected glutathione metabolism genes were demonstrated in susceptible *rbohD*–TuMV and resistant *rbohF*– and *rbohD/F*–TuMV interactions. The presented results of GSH localization with quantification indicated apoplast association, especially in resistance reaction. Therefore, we examined the relative expression level of *AtGGT1* gene in resistant and susceptible *rbohF-*, *rbohD-*, and *rbohD/F* mutants during TuMV interactions. The apoplastic glutathione pool is degraded by GGTs [E.C. 2.3.2.2], which cleave the γ-glutamyl moiety of GSH and GSSG [60,61,62]. *Arabidopsis* genome encodes four *GGT* genes, but only *AtGGT1* is expressed in all plant tissues, but predominantly in leaves and the vascular system [63,64]. Finally, *AtGGT1* encoding product, which is localized in apoplast, where it participates in the degradation of GSSG and the recycling of its amino acids which are then used in the synthesis of GSH in symplast [65]. As reported by Masi et al. [65], these amino acids can be translocated to the cytosol for protein synthesis or resynthesis of GSH through round export degradation in the apoplast. In our experiment, we observed the most dynamic and steady induction of the relative expression of *AtGGT1* in *rbohD/F*–TuMV and *rbohF*–TuMV interaction. These findings indicate that *AtGGT1* plays a role in redox signaling from apoplast to internal compartments in symplast. Moreover, the analysis of GGT activity clearly indicated that almost all activity was associated with the apoplast region and was highly upregulated during the most resistant *rbohD/F*–TuMV interaction and resistant *rbohF*–TuMV interaction. Although virus inoculation activated GGT at 3-dpi timepoint, the enzyme activity was dynamically downregulated between 3 and 14 dpi during susceptible interaction in *rbohD* and Col-0. Our findings on resistance reaction are consistent with those of Ohkama-Ohtsu et al. [66], who postulated that GGT1 metabolizes extracellular GSSG to protect from oxidative stress. The activity of GGT was higher in apoplast, while the lower GSSG content was noted in apoplast especially between 7 and 14 dpi.

Several studies have investigated the potential role of GSTs, especially one of the largest Arabidopsis enzymes *class tau*, but our knowledge regarding their contribution in plant–virus interactions still remains poor. In our experiment, normalized relative expression levels of selected *AtGSTU* genes were found to be significantly changed in susceptible *rbohD*–TuMV and Col-0–TuMV as well as resistant *rbohF*– and *rbohD/F*–TuMV interactions. Moreover, in susceptible Col-0–TuMV and *rbohD*–TuMV interactions, where virus infection was developed, a highly dynamic induction of *AtGSTU1* and *ATGSTU24*. On the other hand, *AtGSTU13* and *AtGSTU19* were downregulated especially at 7–14 dpi. Furthermore, *AtGSTU13* was significantly induced only till symptoms development—up to 7 dpi, as was reported by Otulak-Kozieł et al. [40]. Therefore, we assume that *AtGSTU1* and *AtGSTU24* play a role in susceptible reaction to TuMV, and most importantly in enhanced susceptible *rbohD*–TuMV interaction. In contrast, AtGSTU13 and AtGSTU19 were the most dynamically correlated with resistant *rbohF*–TuMV interaction, and especially enhanced resistant *rbohD/F*–TuMV interaction. The differentially regulated relative expression of *AtGSTU* genes was strictly associated with the modulation of glutathione levels and was correlated with the activity of total GSTs pool. The activity of GSTs was intensively induced during resistant *rboh*–TuMV reactions, and the most in *rbohD/F*–TuMV reaction, whereas in susceptible Col-0–TuMV and *rbohD*–TuMV reactions, their activity decreased. Some individual and limited information about the engagement of certain GSTUs in plant–pathogen interactions is available. The role of GSTU13 in another pathosystem in *Glycine max* was reported by Zhang et al. [67] in terms of the plant–virus interaction. These authors suggested the contribution of GSTUs in the induction of symptoms development at transcriptional and protein level during *Soybean mosaic virus* infection. Piślewska-Bednarek et al. [68] documented that GSTU13 plays a role in GSH conjugation, inducing an immune response in *A. thaliana* against fungal pathogens. Moreover, Pavan Kumar et al. [69] highlighted the accumulation of GST proteins during systemic infection in susceptible interaction between soybean and *Mungbean yellow mosaic virus* as well as *Mungbean yellow mosaic Indian virus*. Furthermore, some GSTU representatives were also documented in plant–virus interactions. Chen et al. [70] confirmed that *GSTU4* from *N. benthamiana* upregulated the plant interactions with *Bamboo mosaic virus*. Méndez-López et al. [71] reported that *S. lycopersicon GSTU38* functioned as a *Pepino mosaic virus* (PepMV) susceptibility factor. Moreover, these authors postulated the dual role of proviral SlGSTU38 in interaction with PepMV capsid protein, and indicated that it may also delay PepMV infection sensing by participating in redox intracellular homeostasis in a nonspecific manner. On the other hand, soybean *GSTU10-10* was identified to be specifically induced after systemic infection by SMV. There are also a few reports indicating the role of selected induced GSTUs in resistance reactions. Kiraly et al. [72] observed increased *NtGSTU1* between 3 and 6 h after TMV inoculation, accompanied by limited virus replication process. Moreover, Satoh et al. [73] confirmed that all GST genes were upregulated in the resistance response of rice to *Rice tungro spherical virus*. Furthermore, Wang et al. [74] postulated that overexpression of *Triticum aestivum GSTU6* in Arabidopsis induced resistance response to *P. syringae* pv. Tomato DC300. In contrast to our observations, Chaouch et al. [17] reported that in *rboh* mutants (*rbohF* and *rbohD* single mutants), no significant changes in the relative expression of *GSTU24* to actin were found, despite the accumulation of a higher level of glutathione in *rbohF* than *rbohD* mutants in response to *P. syringae.*

These findings indicate that different GSTU genes can be differentially expressed in various pathosystems, resulting in differential regulation of oxidized and reduced glutathione content accompanied by changes in the activity of glutathione metabolism enzymes. Similar to our observations, Wu et al. [75] observed induced activity of GST in response to *Sugarcane mosaic virus* (ScMV) during interaction with sorghum plants. These authors noted increased GST activity at 3-dpi timepoint of resistance reaction, but in susceptible plant infected with ScMV, the enzyme activity was significantly decreased. In our experiment, strong induction of GST activity was correlated with resistance response in *rbohF*– and especially *rbohD/F*–TuMV interactions. Díaz-Vivancos et al. [76] also postulated that systemic infection of *Plum pox virus* was correlated with the downregulation of GST activity. This finding is in line with our observations as well as with the decrease of total GR activity. Similarly, Amari et al. [77] observed a significant decrease in GR in the *Prune necrotic ringspot virus* (PNRSV)–apricot interaction, while Clarke et al. [78] made the same observation in susceptible *White clover mosaic virus*–bean interactions. Moreover, compared to that observed in our previous research, resistant *rbohF*– and *rbohD/F*–TuMV interactions, similar to *Atgstu24*–TuMV interaction, resulted in higher induction of GR activity [40]. In contrast, *rbohD*– and Col-0–TuMV interactions, similar to the susceptible *Atgstu19*–TuMV interaction, significantly downregulated GR activity. Therefore, it can be postulated that resistance reaction displayed by *rbohF* and *rbohD/F* mutants was associated with the induction of cellular and apoplastic GR activity. Moreover, similar to GGT activity, GR activity was induced only until 7-dpi timepoint in susceptible responses. Therefore, it can be assumed that GST and GR can contribute in symptoms development and the establishment of reaction. Furthermore, the activity of these enzymes in *rbohD*–TuMV pathosystem was even lower compared to Col-0 plants.

Glutathione peroxidases (GPXs) [E.C.1.11.1.9, E.C. 1.11.1.12, E.C. 1.11.1.15] play a pivotal role in peroxide detoxification [79,80,81]. Plant GPXs are closely related to mammalian ones than to fungal GPXs; thus, they are named “glutathione peroxidase-like enzymes”. GPXLs were assumed to act as a link between glutathione-based and tioredoxin (TRX)-based detoxifying system but they can also use glutathione [82]. Moreover, as reported by Riyazuddin et al. [83,84], plant GPXLs not only protect cell from stress-induced oxidative damage, but also take part in the regulation process associated with development and plant growth. AtGPXLs may function both as ROS scavengers and as redox transducers, and can link GSH with the TRX redox system. Analysis of *GPXLs* overexpression showed enhanced tolerance especially under abiotic stress, but decreased resistance under biotic stress [85,86]. In our experiment, the activity of AtGPXLs was found to be significantly downregulated in resistant *rbohD/F*–TuMV as well as *rbohF*–TuMV interactions. Moreover, the increase of GPXLs activity was accompanied by the induction of lipid peroxidation in susceptible Col-0-TuMV and especially in *rbohD*–TuMV interactions. Therefore, lower GPXLs activity was associated with lower lipid peroxidation in resistant plants compared to susceptible *rbohd*– and Col-0–TuMV interactions. Furthermore, Igbal et al. [87] and Navrot et al. [88] pointed out that AtGPXLs efficiently reduced lipid peroxides and plasma membrane-localized AtGPXLs play an important role in maintaining membrane integrity. Similar to our reports, Singh et al. [56] stated that GPX activity was more dynamically induced in a susceptible cultivar during *Yellow mosaic virus* infection at 20 dpi. Additionally, in contrast to our findings, Kalapos et al. [89] reported high induction of GST genes accompanied by markedly suppressed GPXL, based on the results of transcriptome profiling during ObPV–*C. annuum* HR. Considering these findings, it can be stated that abiotic stress reaction as well as increased activity of antioxidant enzymes, such as ascorbate peroxidase, GR, and GPXL, is associated with reduced levels of stress markers and MDA [90].

It can be summarized that susceptible *rbohD*–TuMV and Col-0–TuMV reactions resulting from systemic virus infection were associated with reduced total glutathione localization and content between 7 and 14 dpi. GSH content increased only up to 7 dpi, when symptoms were established, while the apoplastic level of GSSG dynamically increased between 1 and 14 dpi. In susceptible response, glutathione metabolism was highly correlated with the induction of *AtGSTU1* and *AtGSTU24* genes, which was followed by the decrease of GST and cellular and apoplastic GGT with GR activities between 7 and 14 dpi. In contrast, significant upregulation of GPXL activity was associated with a high rate of lipid peroxidation.

On the contrary, different scenarios were observed in resistant *rbohF*–TuMV and especially enhanced *rbohD/F*–TuMV interaction, which revealed significant reduction of virus expression as well as highly dynamic increase of glutathione localization in all leaf tissues with cellular and apoplastic total glutathione content. The resistant reactions were associated with the upregulation of *AtGGT1*, *AtGSTU13*, and *AtGSTU19* genes, whereas *AtGSTU1* participated in symptoms development at the 7-dpi timepoint. The relative expression of these genes and glutathione changes in resistance reactions were followed by highly dynamic increase of GSTs, and cellular as well as apoplastic GR with GGT activities. The presented results clearly indicate that glutathione can act as a significant factor in susceptible *rbohD* transposon *Arabidopsis* mutant and in resistant *rbohF* and *rbohD/F* mutants during TuMV interaction.

## 4. Materials and Methods

### 4.1. Plant Material, Virus Inoculation, and DAS-ELISA and Molecular Test for TuMV Content

Changes occurring during viral infections induced by TuMV and related to glutathione content were examined in *A. thaliana* (L.) Heynh wild-type (Col-0) plants and specific mutants—*A. thaliana rbohD* (*rbohD*, knockout mutant carrying a single dSpm transposon insertion in *RbohD*), *A. thaliana rbohF* (*rbohF*, knockout mutant carrying a single dSpm transposon insertion in *RbohF*), and *A. thaliana rbohD/F* (*rbohD/F*, double knockout mutant obtained by crossing *rbohF* and *rbohD* single mutants) [48]. All homozygous mutant seeds were kindly provided by the Miguel-Angel Torres Laboratory. The plants were sown on peat rings in pods and grown in growth chamber photon flux density of 100 µmol m^−2^ s ^−1^ (10/14 day/night period), at a relative humidity of 70% and 21 °C. For inducing TuMV inoculation, 19-day-old plants without any lesions and/or alterations were used. The Col-0 and mutant Arabidopsis plants were Wlash inoculated as described by Otulak-Kozieł et al. [40], Tomilson [91], and Walsh and Jenner [92] using the TuMV inoculum (isolate PV-0104 was kindly provided by the Leibniz Institute, Braunschweig, Germany) in phosphate buffer [93]. The leaves of mock- and TuMV-inoculated Col-0 and mutant plants were validated for the presence of virus using qPCR (quantitative polymerase chain reaction). The level of TuMV was determined by performing qPCR analysis of the *TuMV-CP* gene fragment using the primers presented by Arous et al. [94] according to the procedure of Otulak-Kozieł et al. [40]. *TuMV-CP* expression was compared with the mean expression of the plant host reference genes, *AtEf1α* and *AtF-Box*, as described by Otulak-Kozieł et al. [40]. A total of 60 plants (30 virus-inoculated and 30 mock-inoculated Col-0 and mutant plants of different types) were used for determining *TuMV-CP* expression and for other analyses (immunofluorescence microscopy, CTCF estimation, HPLC, *AtGSTUs* and *AtGGT1* gene expression profiling, determination of GST/GR/GPXL enzymatic activity). All the analyses were performed in triplicate using a new set of plants every time.

### 4.2. Quantification of Immunofluorescence and Immunogold Localization of Total Glutathione in TuMV-Inoculated Col-0, rbohD, rbohF, and rbohD/F Arabidopsis Leaves

Mock- and TuMV-inoculated Col-0, *rbohD*, *rbohF*, *and rbohD/F* leaves were collected, fixed, and treated as described by Kozieł et al. [95] and Kozieł et al. [96] for immunofluorescence localization. To validate the distribution of total glutathione, quantification of immunolocalization based on CTCF (corrected total cell fluorescence) was performed according to the procedure of Kozieł et al. [97] without DAPI (4′,6-diamidino-2-phenylindole) staining, as described by Otulak-Kozieł et al. [98]. For detection of glutathione content in immunofluorescent localizations, primary polyclonal rabbit antibodies targeting the total glutathione content (1:100 dilution; Merck, Darmstadt, Germany, catalog number: AB5010) were used as described by Otulak-Koziel et al. [40]. Visualization was performed using respectively secondary anti-rabbit antibodies conjugated with AlexaFluor^®^488 (Jackson ImmunoResearch Europe Ltd., Cambridgeshire, UK, catalog number: 111-545-144). Slides with leaf fragments were imaged on a PROVIS AX70 fluorescence microscope with an Olympus UP90 high-definition camera (Olympus, Warsaw, Poland) using Olympus Cell Sense Standard Software (version 1.18; Olympus, Center Valley, PA, USA). The strength of green fluorescence signal indicating total glutathione localization was validated using a quantitative measuring method, CTCF, as previously described [97,98] with one modification. CTCF was measured for whole cells and separately for apoplast (CTCF apop) regions of the cell. For each combination the cell CTCF and CTCF apop, 30 selected areas of each sample were analyzed based on 50 photos. The levels of fluorescence signal were measured using Fiji (version 2.9.0/1.53 t; National Institutes of Health, Bethesda, MD, USA). Measurements of green immunofluorescence signals obtained using Fiji were calculated to determine CTCF or CTCF apop at 20× magnification with 1.00 zoom factor using a formula previously presented by Otulak-Kozieł et al. [24]. The estimated cell CTCF and CTCF apop values were then analyzed statistically at selected time intervals for all plants by one-factor analysis of variance (ANOVA) [24].

Parallel to immunofluorescence we performed the quantified immunogold localization of total glutathione as was described in [40]. Mock- and TuMV-inoculated Col-0, *rbohD*, *rbohF*, and *rbohD/F* leaves were collected, fixed, and treated as described by Kozieł et al. [40]. For detection of glutathione content, we used the same type of primary polyclonal rabbit antibodies targeting the total glutathione content as in immunofluorescence (1:100 dilution; Merck, Darmstadt, Germany, catalog number: AB5010) [40]. Visualization was performed using secondary antirabbit antibodies conjugated with anti-rabbit antibodies conjugated with 18 nm nanogold particles (Jackson ImmunoResearch Europe Ltd., Cambridgeshire, UK, catalog number: 711-215-152). The immunogold-labeled sections on the grids were examined using a transmission electron microscope [99]. Then, labeling was quantified following the method of Luschin-Ebengreuth and Zechmann [100] in specific cell sections in the case of glutathione and globally in the case of TuMV. Statistical analyses were performed, as described by Otulak-Kozieł et al. [99]. The concentrations of gold particles in specific cell sections and globally were validated using ANOVA and post hoc Tukey’s HSD (honestly significant difference) test using Statistica software (version 13.0; StatSoft and TIBCO Software Inc., Palo Alto, CA, USA). ANOVA was used to estimate gold labeling. For the statistical estimation of immunogold labeling, infected and mock-inoculated materials were compared at the 7 and 14 dpi time point. The number of gold particles globally or in-cell compartments was counted in 35 fields (10 μm^2^) per image. For each combination (mock-inoculated plants and TuMV-inoculated Col-0 and mutant plants), gold particles from 200 photographs were counted to determine the presence of glutathione or TuMV content.

### 4.3. HPLC Analysis of Cellular and Apoplastic Content of GSH, GSSG, and Total Glutathione Accompanied by Estimation of Cytoplasmic Contamination

The cellular and apoplastic GSH, GSSG, and total glutathione (GSH+GSSG) contents in mock- and TuMV-inoculated Col-0, *rbohD*, *rbohF*, and *rbohD/F* plants were measured by reversed-phase HPLC with fluorescence detection, as reported by Kranner [101], using the exact procedure presented by Otulak-Kozieł et al. [24]. Briefly, leaf samples for HPLC were collected as described by Otulak-Kozieł et al. [25]. Some parts of samples were used directly for HPLC analyses [101] and for estimating the activity of leaf glucose-6-phosphate dehydrogenase (G6PDH; EC 1.1.1.49), while other parts were used for the extraction of apoplastic components (apoplastic washing fluid, AWF), and then G6PDH activity was determined and HPLC analyses were performed, respectively, as described by Borniego et al. [102] and Ohkama-Ohtsu et al. [41]. The extraction of apoplastic components was performed according to the procedure of Vanacker et al. [103,104] with modifications of Ohkama-Ohtsu et al. [41]. For this, a solution consisting of 50 mM MES-KOH buffer (pH 6.0), 40 mM KCl, and 2 mM CaCl_2_ was vacuum-infiltered into leaves. After recovering the apoplastic solution (AWF) by centrifugation at 2900× *g*, the solution was divided into two parts. One was used for measuring the G6PDH activity, which was used as a marker for estimating the cytoplasmic contamination of samples according to the procedures of Weimar and Rothe [105] and Vanacker et al. [103,104]. The second part was used for determining GSH and GSSG by HPLC after sample deproteination with an equal volume of 10% metaphosphoric acid. G6PDH activity was estimated in AWF and leaf exactly as described by Borniego et al. [102] at room temperature in a 100 µL solution containing 100 mM Tris-HCl (pH 8), 6.7 mM MgCl_2_, 12 mM glucose-6-phosphate, 0.4 mM NADP, and AWF or leaf extracts obtained from all types of plants at 1, 3, 7, and 14 dpi. NADP reduction was followed at 340 nm. The activity was estimated in unit (U), where 1 U is equal to 1 nmol of reduced NADP/min/mg total protein. After the estimation of G6PDH activity in AWF and leaves, we compared the values to validate cytoplasmic contamination of AWF, as described by Ohkama-Ohtsu et al. [43]. It was assumed that if the ratio of G6PDH activity in AWF to that in total leaf extracts was less than 0.25%, then there was no statistically significant cytoplasmic contamination (Table S4). GSH, GSSG, and GSH+GSSG contents were estimated using the results of standards and presented as nmol g^−1^ FW. The ratio of GSH/GSSG was calculated for particular combination of TuMV infected Col-0 and mutant plants.

For HPLC analyses of G6PDH activity, a total of 60 plants were used (30 virus-inoculated and 30 mock-inoculated Col-0 plants and mutant plants of all three types). All analyses were performed in triplicate using a new set of plants every time. The cellular and apoplastic GSH, GSSG, and GSH+GSSG concentrations were analyzed statistically at selected time intervals for all plants by ANOVA.

### 4.4. Isolation of RNA and Genomic DNA (gDNA) for AtGGT1 and Selected AtGSTU Genes in TuMV-Infected Col-0, rbohD, rbohF, and rbohD/F Plants

To estimate the expression of *A. thaliana GSTU* genes in the plant host, molecular analyses were performed on the samples collected at 3, 7, and 14 dpi. Briefly, leaf samples (0.1 g of each sample) were collected from 30 mock- (buffer) or virus-infected Col-0 plants and mutant plants of different types. Analyses of RNA isolation, purification, and quality as well as confirmation of lack of RNA contamination were performed following previously described procedures [24,40]. The absence of RNA contamination was also verified again by performing reverse transcription PCR using *AtEf1α* and *AtF-Box* as reference standards [40], which confirmed the absence of contaminating gDNA. Then, cDNA was synthesized using the NG dART RT Kit (EURx Sp. z o.o., Gdansk, Poland), according to the manufacturer’s instructions. Reverse transcription reactions were performed as described by Otulak-Kozieł et al. [40].

### 4.5. Analysis of Relative Expression of AtGGT1 and Selected AtGSTU Genes in TuMV-Infected Col-0, rbohD, rbohF, and rbohD/F Plants Using qPCR

Real-time qPCR was performed using the Bio-Rad CFX96Touch^TM^ apparatus (Bio-Rad, Hercules, CA, USA) and Fast SG qPCR Master Mix (2x) (EURx Sp. z o.o., Gdansk, Poland), as previously described by Otulak-Kozieł et al. [40] for *AtEf1α* and *AtF-Box* as reference genes. All qPCR tests were calibrated using previously prepared five-point calibration curves (based on cDNA and gDNA). The analyzed *A. thaliana* GSTU genes *GSTU1* (*AtGSTU1*, AT2G29490), *GSTU13* (*AtGSTU13*, AT1G27130), *GSTU19* (*AtGSTU19*, AT1G78380), and *GSTU24* (*AtGSTU24*, AT1G17170) were selected based on involvement in TuMV and other pathogen host reaction [40,68,106]. *AtGGT1* (γ-glutamyl transpeptidases gene, AT4G39640) was selected because of its location and role in GSH catabolism in cell wall. *AtGGT1*, *AtGSTU1*, *AtGSTU13*, *ATGSTU19*, and *AtGSTU24* were analyzed by qPCR in comparison to reference genes. The expression of these genes in *A. thaliana* was validated, and complete sequences were acquired from the TAIR database [107]. Based on previously published papers, the primers were chosen for *AtGGT1* [41] and *AtGSTU* [40]. All the primers used in the experiments are shown in Table S5. The starting cDNA solution (used for generating calibration curves) was a four-fold-diluted mix of 10 randomly selected cDNA mixes. An eight-fold-diluted cDNA mix was used to construct the calibration curve for gDNA, while the subsequent calibration points were measured at fourfold dilutions in a 15-µL volume. A 5-µL solution of eight-fold-diluted cDNA mix was added to the reaction mixture. The conditions used for qPCR analyses are presented in Table S6. The molecular analyses were performed on 60 plants (30 virus-inoculated and 30 mock-inoculated Col-0 and mutant plants of all types) in triplicate using a new set of plants every time. The expression was analyzed statistically at selected time intervals for all plants by ANOVA.

### 4.6. Validation of GST, GR, GPXL, and GGT Activities and Lipid Peroxidation in Leaves of TuMV-Infected Col-0, rbohD, rbohF, and rbohD/F Plants

To validate the activity of gluthatione S-transferase (GST, EC 2.5.1.18), gluthatione reductase (GR, EC 1.8.1.7), gluthatione peroxidase like (GPXL, EC 1.11.1.9), and γ-glutamyl transpeptidase (GGT, EC 2.3.2.2) in Col-0, *rbohD*, *rbohF*, and *rbohD/F* plants, the leaves of these plants were collected at 3, 7, and 14-dpi timepoints after the inoculation of mock or TuMV. A total of 60 plants (30 virus-inoculated and 30 mock-inoculated Col-0 and mutant plants of all three types) were used. All analyses were performed in triplicate using a new group of plants every time. The activity of GST was validated as described by Islam et al. [108] and Otulak-Kozieł [39], and determined based on the enzyme’s ability to conjugate GSH and 1-chloro-2,4-dinitrobenzene (CDNB) at 344 nm [109]. The results were presented in U, where 1 U is equal to 1 µmol of CDNB conjugated/min/mg total protein. The activity of GR was determined by measuring the increase in absorbance at 412 nm when 5,5′-dithio-bis(2-nitrobenzoic acid) (DTNB) was reduced by GSH, generated from GSSG, as proposed by Bela et al. [110]. The enzyme activity was calculated as the amount of reduced DTNB in U, where 1 U is equal to 1 µmol of DTNB conjugated/min/mg total protein (ε420 = 13.6 mM^−1^ cm^−1^). The activity of GPXLs in leaves was also measured spectrophotometrically using cumene hydroperoxide (CHP; Sigma-Aldrich, Darmstadt, Germany) as substrate, as described by Riyazuddin et al. [83]. The compositions of GPXL reaction mixtures were as follows: 4 mM GSH, 0.2 mM NADPH, 0.05 U of GR (from baker’s yeast; Sigma-Aldrich, Darmstadt, Germany), 100 μL enzyme extract, and 0.5 mM substrate in a phosphate buffer (0.1 M, pH 7.0) in a total volume of 1 mL. GPXL activity was expressed in U, where 1 U is equal to 1 nmol of converted NADPH/min/mg total protein (ε340 = 6.22 mM^−1^ cm^−1^). For determining GGT activity, extracts from mock- and virus-inoculated leaves were prepared using a modified version of the protocol described by Martin and Slovin [61] with modifications presented by Ohkama-Ohtsu et al. [41]. Briefly, 200 mg of leaf tissue was ground in 400 µL of a buffer containing 1 mM NaCl, 100 mM Tris-HCl (pH 8.0), 1 mM benzamidine, 1 mM 6-amino-n-hexanoic acid, 1 mM phenylmethylsulfonyl fluoride, 1 mM leupeptin, and 0.1% Triton X-100. During extraction, 1 mg of polyvinylpolypyrrolidone (PVPP) was added, and the resulting mixture was incubated on ice for 5 min and then centrifuged at 16,000× *g* for 15 min at 4 °C. The obtained supernatant was used to determine GGT activity which was measured spectrophotometrically at 410 nm using γ-GPNA (Sigma-Aldrich, Darmstadt, Germany) as substrate and glycyl-glycine (Sigma-Aldrich, Darmstadt, Germany) as acceptor. The determination was performed as described by Tate and Meister [111] and Ohkama-Ohtsu et al. [41] in a 1 mL solution containing 100 mM Tris-HCl (pH 8.0), 5 mM γ-GPNA, and 100 mM glycyl-glycine, with protein added for initiating the reaction. The enzyme activity was measured over a 10-min interval. In accordance with Ohkama-Ohtsu et al. [41], 1 U of GGT activity was assumed to be equal to the formation of 1 nmol p-nitroanilide/min/mg total protein [41]. To check the modulation of the activity of glutathione-associated enzymes, lipid peroxidation in leaf tissues was validated in mock- and virus-inoculated plants at 7 and 14 dpi, as described by Burian et al. [112] and according to the methodology proposed by Hoges et al. [42]. Lipid peroxidation was determined based on the level of the specific reaction product malondialdehyde (MDA). MDA content was measured in the presence of thiobarbituric acid (TBA, Sigma-Aldrich, Darmstadt, Germany). Control reactions were simultaneously performed without the addition of TBA. The absorbance of each sample was measured at λ = 440 nm, 532 nm, and 600 nm to correct nonspecific product contamination. The results of lipid peroxidation were presented as µmol MDA/g FW. All enzymatic activities and lipid peroxidation were analyzed statistically at selected time intervals for all plants by ANOVA.

### 4.7. Validation of Apoplastic GR and GGT Activities in TuMV-Infected Col-0, rbohD, rbohF, and rbohD/F Leaves

Apoplastic solutions were extracted according to the procedure of Ohkama-Ohtsu et al. [41] from mock inoculated Col-0 and mutant plants without the deproteination step. Validation of GR and GGT activities was performed as presented in Section 4.6.

### 4.8. Estimation of PCCs for Elements of Host Reaction

To validate the relationship between cell and apoplast, correlation analyses were performed with the use of Pearson’s correlation coefficients (PCCs). We compared the likelihood with levels of TuMV for the following variables: cellular/apoplastic total glutathione localization, cellular/apoplastic content of GSH/GSSG, expression of host genes (*AtGGT1*, *AtGSTU1*, *AtGSTU13*, *AtGSTU19*, *AtGSTU24*), and cellular/apoplastic glutathione-associated enzyme activity. Pairwise correlations between the abovementioned variables and the levels of TuMV were determined at 7 and 14 dpi in Col-0 and all mutant plants as described by Wu et al. [113] and Manders et al. [114] using Excel 2019 software (Microsoft, Poland, Warsaw). The results were presented in the form of a heat map generated using the PCC values, where values higher than 0.70 were considered to reflect a strong positive correlation between the analyzed pairs.

## Data Availability

Not applicable.

## Supplementary Materials

The following supporting information can be downloaded at: https://www.mdpi.com/article/10.3390/ijms24087128/s1.

## References

[1] Suzuki N., Miller G., Morales J., Shulaev V., Torres M.A., Mittler R. (2011). Respiratory burst oxidases: The engines of ROS signaling. Curr. Opin. Plant Biol..

[2] Oda T., Hashimoto H., Kuwabara N., Akashi S., Hayashi K., Kojima C., Wong H.L., Kawasaki T., Shimamoto K., Sato M. (2010). Structure of the N-terminal regulatory domain of a plant NADPH oxidase and its functional implications. J. Biol. Chem..

[3] Wang W., Chen D., Zhang X., Liu D., Cheng Y., Shen F. (2018). Role of plant respiratory burst oxidase homologs in stress responses. Free Radic. Res..

[4] Sagi M., Fluhr R. (2006). Production of reactive oxygen species by plant NADPH oxidases. Plant Physiol..

[5] Groom Q.J., Torres M.A., Fordham-Skelton A.P., Hammond-Kosack K.E., Robinson N.J., Jones J.D. (1996). rbohA, a rice homologue of the mammalian gp91phox respiratory burst oxidase gene. Plant J..

[6] Yu S., Kakar K.U., Yang Z., Nawaz Z., Lin S., Guo Y., Ren X.L., Baloch A.A., Han D. (2020). Systematic study of the stress-responsive *Rboh* gene family in Nicotiana tabacum: Genome-wide identification, evolution and role in disease resistance. Genomics.

[7] Zhang J., Xie Y., Ali B., Ahmed W., Tang Y., Li H. (2021). Genome-wide Identification, Classification, Evolutionary Expansion and Expression of *Rboh* Family Genes in Pepper (*Capsicum annuum* L.). Trop. Plant Biol..

[8] Liu J., Lu H., Wan Q., Qi W., Shao H. (2018). Genome-wide analysis and expression profiling of respiratory burst oxidase homologue gene family in *Glycine max*. Environ. Exp. Bot..

[9] Zhang Y., Li Y., He Y., Hu W., Zhang Y., Wang X., Tang H. (2018). Identification of NADPH oxidase family members associated with cold stress in strawberry. FEBS Open Bio..

[10] Li D., Wu D., Li S., Dai Y., Cao Y. (2019). Evolutionary and functional analysis of the plant-specific NADPH oxidase gene family in *Brassica rapa* L.. R. Soc. Open Sci..

[11] Huang S., Tang Z., Zhao R., Hong Y., Zhu S., Fan R., Ding K., Cao M., Luo K., Geng M. (2021). Genome-wide identification of cassava MeRboh genes and functional analysis in Arabidopsis. Plant Physiol. Biochem..

[12] Hu C.-H., Wang P.-Q., Zhang P.-P., Nie X.-M., Li B.-B., Tai L., Liu W.-T., Li W.-Q., Chen K.-M. (2020). NADPH Oxidases: The Vital Performers and Center Hubs during Plant Growth and Signaling. Cells.

[13] Zandalinas S.I., Fichman Y., Mittler R. (2020). Vascular bundles mediate systemic reactive oxygen signaling during light stress. Plant Cell.

[14] Morales J., Kadota Y., Zipfel C., Molina A., Torres M.A. (2016). The *Arabidopsis* NADPH Oxidases *Rbohd* and *Rbohf* Display Differential Expression Patterns and Contributions During Plant Immunity. J. Exp. Bot..

[15] Torres M.A., Jones J.D., Dangl J.L. (2005). Pathogen-induced, NADPH oxidase-derived reactive oxygen intermediates suppress spread of cell death in Arabidopsis thaliana. Nat. Genet..

[16] Proels R.K., Oberhollenzer K., Pathuri I.P., Hensel G., Kumlehn J., Huckelhoven R. (2010). Rbohf2 of Barley Is Required for Normal Development of Penetration Resistance to the Parasitic Fungus *Blumeria graminis* F. sp. Hordei. Mol. Plant-Microbe Interact..

[17] Chaouch S., Queval G., Noctor G. (2012). Atrbohf Is a Crucial Modulator of Defence-Associated Metabolism and a Key Actor in the Interplay between Intracellular Oxidative Stress and Pathogenesis Responses in *Arabidopsis*. Plant J..

[18] Yoshioka H., Numata N., Nakajima K., Katou S., Kawakita K., Rowland O., Jones J.D.G., Doke N. (2003). Nicotiana Benthamiana gp91^(Phox)^ Homologs Nbrboha and Nbrbohb Participate in H_2_O_2_ Accumulation and Resistance to Phytophthora Infestans. Plant Cell.

[19] Zhang Z., van Esse H.P., van Damme M., Fradin E.F., Liu C.M., Thomma B.P. (2013). Ve1-mediated resistance against Verticillium does not involve a hypersensitive response in Arabidopsis. Mol. Plant Pathol..

[20] Zhao J., Chen Q., Zhou S., Sun Y., Li X., Li Y. (2020). H2Bub1 Regulates *RbohD*-Dependent Hydrogen Peroxide Signal Pathway in the Defense Responses to *Verticillium ahlia* Toxins. Plant Physiol..

[21] Trujillo M., Altschmied L.., Schweizer P., Kogel K.H., Huckelhoven R. (2006). Respiratory burst oxidase homologue A of barley contributes to penetration by the powdery mildew fungus *Blumeria graminis* f. sp. Hordei. J. Exp. Bot..

[22] Foley R.C., Gleason C.A., Anderson J.P., Hamann T., Singh K.B. (2013). Genetic and Genomic Analysis of Rhizoctonia Solani Interactions with *Arabidopsis*; Evidence of Resistance Mediated through NADPH Oxidases. PLoS ONE.

[23] Doke N., Ohashi Y. (1988). Involvement of an O_2_^−^ generating system in the induction of necrotic lesions on tobacco leaves infected with tobacco mosaic virus. Physiol. Mol. Plant Pathol..

[24] Otulak-Kozieł K., Kozieł E., Valverde R.A. (2019). The respiratory burst oxidase homolog d (*rbohd*) cell and tissue distribution in potato–Potato virus Y (PVY^ntn^) hypersensitive and susceptible reactions. Int. J. Mol. Sci..

[25] Otulak-Kozieł K., Kozieł E., Bujarski J.J., Frankowska-Łukawska J., Torres M.A. (2020). Respiratory Burst Oxidase Homologs RBOHD and RBOHF as Key Modulating Components of Response in Turnip Mosaic Virus—*Arabidopsis thaliana* (L.) Heyhn System. Int. J. Mol. Sci..

[26] Pei Z.M., Murata Y., Benning G., Thomine S., KluÈsener B., Allen G.J., Grill E., Schroeder J.I. (2000). Calcium Channels Activated by Hydrogen Peroxide Mediate Abscisic Acid Signalling in Guard Cells. Nature.

[27] Boudsocq M., Willmann M.R., McCormack M., Lee H., Shan L., He P., Bush J., Cheng S.H., Sheen J. (2010). Differential Innate Immune Signalling via Ca^2+^ Sensor Protein Kinases. Nature.

[28] Lin F., Ding H., Wang J., Zhang H., Zhang A., Zhang Y., Tan M., Dong W., Jiang M. (2009). Positive Feedback Regulation of Maize NADPH Oxidase by Mitogen-Activated Protein Kinase Cascade in Abscisic Acid Signalling. J. Exp. Bot..

[29] Yun B.W., Feechan A., Yin M., Saidi N.B., Bihan T.L., Yu M., Moore J.W., Kang J.G., Kwon E., Spoel S.H. (2011). S-Nitrosylation of NADPH Oxidase Regulates Cell Death in Plant Immunity. Nature.

[30] Cairns N.G., Pasternak M., Wachter A., Cobbett C.S., Meyer A.J. (2006). Maturation of *Arabidopsis* seeds is dependent on glutathione biosynthesis within the embryo. Plant Physiol..

[31] Noctor G., Queval G., Mhamdi A., Chaouch S., Foyer C.H. (2011). Glutathione. Arab. Book.

[32] Foyer C.H., Noctor G. (2011). Ascorbate and glutathione: The heart of the redox hub. Plant Physiol..

[33] Foyer C.H., Theodoulou F.L., Delrot S. (2001). The functions of inter- and intracellular glutathione transport systems in plants. Trends Plant Sci..

[34] Sabetta W., Paradiso A., Paciolla C., de Pinto M.C., Hossain M.A., Mostofa M.G., Diaz-Vivancos P., Burritt D.J., Fujita M., Tran S.L.P. (2017). Chemistry, biosynthesis, and antioxidative function of glutathione in plants. Glutathione in Plant Growth, Development, and Stress Tolerance.

[35] Diaz-Vivancos P., Wolff T., Markovic J., Pallardó F.V., Foyer C.H. (2010). A nuclear glutathione cycle within the cell cycle. Biochem. J..

[36] Zechmann B., Zellnig G., Urbanek-Krajnc A., Müller M. (2007). Artificial elevation of glutathione affects symptom development in ZYMV-infected Cucurbita pepo L. plants. Arch. Virol..

[37] Gullner G., Tobia I., Fodor J., Kömives T. (1999). Elevation of glutathione level and activation of glutathione-related enzymes affect virus infection in tobacco. Free Radic. Res..

[38] Király L., Albert R., Zsemberi O., Schwarczinger I., Hafez Y.M., Künstler A. (2021). Reactive Oxygen Species Contribute to Symptomless, Extreme Resistance to *Potato virus X* in Tobacco. Phytopathology.

[39] Otulak-Kozieł K., Kozieł E., Przewodowski W., Ciacka K., Przewodowska A. (2022). Glutathione Modulation in PVY^NTN^ susceptible and resistant potato plant interactions. Int. J. Mol. Sci..

[40] Otulak-Kozieł K., Kozieł E., Horváth E., Csiszár J. (2022). AtGSTU19 and AtGSTU24 as Moderators of the Response of *Arabidopsis thaliana* to *Turnip mosaic virus*. Int. J. Mol. Sci..

[41] Ohkama-Ohtsu N., Radwan S., Peterson A., Zhao P., Badr A.F., Xiang C., Oliver D.J. (2007). Characterization of the extracellular gamma-glutamyl transpeptidases, GGT1 and GGT2, in Arabidopsis. Plant J..

[42] Hodges D.M., DeLong J.M., Forney C.F., Prange R.K. (1999). Improving the thiobarbituric acid-reactive-substances assay for estimating lipid peroxidation in plant tissues containing anthocyanin and other interfering compounds. Planta.

[43] Berrocal-Lobo M., Stone S., Yang X., Antico J., Callis J., Ramonell K.M., Somerville S. (2010). ATL9, a RING zinc finger protein with E3 ubiquitin ligase activity implicated in chitin- and NADPH oxidase-mediated defense responses. PLoS ONE.

[44] Lukan T., Pompe-Novak M., Baebler Š., Tušek-Žnidarič M., Kladnik A., Križnik M., Blejec A., Zagorščak M., Stare K., Dušak B. (2020). Precision transcriptomics of viral foci reveals the spatial regulation of immune-signaling genes and identifies RBOHD as an important player in the incompatible interaction between potato virus Y and potato. Plant J..

[45] Pogany M., von Rad U., Grun S., Dongo A., Pintye A., Simoneau P., Bahnweg G., Kiss L., Barna B., Durner J. (2009). Dual Roles of Reactive Oxygen Species and NADPH Oxidase *Rbohd* in an *Arabidopsis*-*Alternaria* Pathosystem. Plant Physiol..

[46] Torres M.A., Dangl J.L., Jones J.D.G. (2002). *Arabidopsis* gp91phox homologues AtrbohD and AtrbohF are required for accumulation of reactive oxygen intermediates in the plant defense response. Proc. Natl. Acad. Sci. USA.

[47] Marino D., Dunand C., Puppo A., Pauly N. (2012). A Burst of Plant NADPH Oxidases. Trends Plant Sci..

[48] Hakmaoui A., Pérez-Bueno M.L., García-Fontana B., Camejo D., Jiménez A., Sevilla S., Barón M. (2012). Analysis of the antioxidant response of *Nicotiana benthamiana* to infection with two strains of *Pepper* mild mottle virus. J. Exp. Bot..

[49] Hernández J.A., Barba E., Diaz-Vivancos P., Hossain M.A., Mostofa M.G., Diaz-Vivancos P., Burritt D.J., Fujita M., Tran S.L.P. (2017). Glutathione-Mediated biotic stress tolerance. Glutathione in Plant Growth, Development, and Stress Tolerance.

[50] Glazebrook J., Rogers E.E., Ausubel F.M. (1996). Isolation of Arabidopsis mutants with enhanced disease susceptibility by direct screening. Genetics.

[51] Parisy V., Poinssot B., Owsianowski L., Buchala A., Glazebrook J., Mauch F. (2007). Identification of PAD2 as a γ-glutamylcysteine synthetase highlights the importance of glutathione in disease resistance of *Arabidopsis*. Plant J..

[52] Ball L., Accotto G.-P., Bechtold U., Creissen G., Funck D., Jimenez A., Kular B., Leyland N., Mejia-Carranza J., Reynolds H. (2004). Evidence for a direct link between glutathione biosynthesis and stress defense gene expression in *Arabidopsis*. Plant Cell.

[53] Kuźniak E., SkŁodowska M. (2004). Differential Implication of Glutathione, Glutathione-Metabolizing Enzymes and Ascorbate in Tomato Resistance to Pseudomonas syringae. J. Phytopathol..

[54] Vanacker H., Carver T.L., Foyer C.H. (2000). Early H2O2 accumulation in mesophyll cells leads to induction of glutathione during the hyper-sensitive response in the Barley-Powdery Mildew interaction. Plant Physiol..

[55] Tolin S., Arrigoni G., Trentin A.R., Veljovic-Jovanovic S., Pivato M., Zechman B., Masi A. (2013). Biochemical and quantitative proteomics investigations in *Arabidopsis* ggt1 mutant leaves reveal a role for the gamma-glutamyl cycle in plant’s adaptation to environment. Proteomics.

[56] Singh Y.J., Grewal S.K., Gill R.K. (2020). Role of glutathione in methylglyoxal detoxification pathway during yellow mosaic virus (YMV) infection in black gram (*Vigna mungo* (L.) Hepper). Physiol. Mol. Plant Pathol..

[57] Künstler A., Király L., Kátay G., Enyedi A.J., Gullner G. (2019). Glutathione can compensate for salicylic acid deficiency in tobacco to maintain resistance to tobacco mosaic virus. Front. Plant Sci..

[58] Király Z., Barna B., Kecskés A., Fodor J. (2002). Down-regulation of antioxidative capacity in a transgenic tobacco which fails to develop acquired resistance to necrotization caused by tobacco mosaic virus. Free Radic. Res..

[59] Han Y., Chaouch S., Mhamdi A., Queval G., Zechmann B., Noctor G. (2013). Functional analysis of Arabidopsis mutants points to novel roles for glutathione in coupling H_2_O_2_ to activation of salicylic acid accumulation and signalling. Antioxid. Redox Signal..

[60] Dorion S., Ouellet J.C., Rivoal J. (2021). Glutathione Metabolism in Plants under Stress: Beyond Reactive Oxygen Species Detoxification. Metabolites.

[61] Martin M.N., Slovin J.P. (2000). Purified gamma-glutamyl transpeptidases from tomato exhibit high affinity for glutathione and glutathione S-conjugates. Plant Physiol..

[62] Storozhenko S., Belles-Boix E., Babiychuk E., Herouart D., Davey M.W., Slooten L., Van Montagu M., Inze D., Kushnir S. (2002). γ-glutamyl transpeptidase in transgenic tobacco plants. Cellular localization, processing, and biochemical properties. Plant Physiol..

[63] Destro T., Prasad D., Martignago D., Bernet I.L., Trentin A.R., Renu I.K., Ferretti M., Masi A. (2011). Compensatory expression and substrate inducibility of gamma-glutamyl transferase GGT2 isoform in *Arabidopsis thaliana*. J. Exp. Bot..

[64] Martin M.N., Saladores P.H., Lambert E., Hudson A.O., Leustek T. (2007). Localization of members of the gamma-glutamyl transpeptidase family identifies sites of glutathione and glutathione S-conjugate hydrolysis. Plant Physiol..

[65] Masi A., Trentin A.R., Agrawal G.K., Rakwal R. (2015). Gamma-glutamyl cycle in plants: A bridge connecting the environment to the plant cell?. Front. Plant Sci..

[66] Ohkama-Ohtsu N., Zhao P., Xiang C.B., Oliver D.J. (2007). Glutathione conjugates in the vacuole are degraded by gamma-glutamyl transpeptidase GGT3 in *Arabidopsis*. Plant J..

[67] Zhang K., Shen Y., Wang T., Wang Y., Xue S., Luan H., Wang L., Li K., Guo D., Zhi H. (2022). GmGSTU13 is related to the development of mosaic symptoms in soybean plants infected with Soybean mosaic virus. Phytopathology.

[68] Piślewska-Bednarek M., Nakano R.T., Hiruma K., Pastorczyk M., Sanchez-Vallet A., Singkaravanit-Ogawa S., Ciesiołka D., Takano Y., Molina A., Schulze-Lefert P. (2018). Glutathione transferase U13 functions in pathogen-triggered glucosinolate metabolism. Plant Physiol..

[69] Pavan Kumar B.K., Kanakala S., Malathi V.G., Gopal M., Usha R. (2017). Transcriptomic and proteomic analysis of yellow mosaic diseased soybean. J. Plant Biochem. Biotechnol..

[70] Chen I.H., Chiu M.H., Cheng S.F., Hsu Y.H., Tsai C.H. (2013). The glutathione transferase of Nicotiana benthamiana NbGSTU4 plays a role in regulating the early replication of Bamboo mosaic virus. New Phytol..

[71] Méndez-López E., Donaire L., Gosálvez B., Díaz-Vivancos P., Sánchez-Pina M.A., Tilsner J., Aranda M.A. (2023). Tomato SlGSTU38 interacts with the PepMV coat protein and promotes viral infection. New Phytol..

[72] Király L., Künstler A., Fattinger M., Höller K., Juhász C., Müller M., Gullner G., Zechmann B. (2012). Sulfate supply influences compartment specific glutathione metabolism and confers enhanced resistance to tobacco mosaic virus during a hypersensitive response. Plant Physiol. Biochem..

[73] Satoh K., Kondoh H., De Leon T.B., Macalalad R.J.A., Cabunagan R.C., Cabauatan P.Q., Mauleon R., Kichuchi S., Choi I.R. (2013). Gene expression responses to Rice tungro spherical virus in susceptible and resistant near-isogenic rice plants. Virus Res..

[74] Wang Q., Guo J., Jin P., Guo M., Guo J., Cheng P., Li Q., Wang B. (2022). Glutathione S-transferase interactions enhance wheat resistance to powdery mildew but not wheat stripe rust. Plant Physiol..

[75] Wu L., Han Z., Wang S., Wang X., Sun A., Zu X., Chen Y. (2013). Comparative proteomic analysis of the plant-virus interaction in resistant and susceptible ecotypes of maize infected with sugarcane mosaic virus. J. Proteom..

[76] Diaz-Vivancos P., Clemente-Moreno M.J., Rubio M., Olmos E., Garcia J.A., Martinez-Gomez P., Hernandez J.A. (2008). Alteration in the chloroplastic metabolism leads to ROS accumulation in pea plants in response to plum pox virus. J. Exp. Bot..

[77] Amari K., Díaz-Vivancos P., Pallás V., Sánchez-Pina M.A., Hernández J.A. (2007). Oxidative stress induction by Prunus necrotic ringspot virus infection in apricot seeds. Physiol. Plant..

[78] Clarke S.F., Guy P.L., Burritt D.J., Jameson P.E. (2002). Changes in the activities of antioxidant enzymes in response to virus infection and hormone treatment. Physiol. Plant..

[79] Attacha S., Solbach D., Bela K., Moseler A., Wagner S., Schwarzländer M., Aller I., Müller S.J., Meyer A.J. (2017). Glutathione peroxidase-like enzymes cover five distinct cell compartments and membrane surfaces in Arabidopsis thaliana. Plant Cell Environ..

[80] Bela K., Bangash S.A.K., Csiszár J. (2017). Plant Glutathione Peroxidases: Antioxidant Enzymes in Plant Stress Responses and Tolerance. Glutathione in Plant Growth, Development, and Stress Tolerance.

[81] Bela K., Riyazuddin R., Csiszár J. (2022). Plant Glutathione Peroxidases: Non-Heme Peroxidases with Large Functional Flexibility as a Core Component of ROS-Processing Mechanisms and Signalling. Antioxidants.

[82] Passaia G., Fonini L.S., Caverzan A., Jardim-Messeder D., Christoff A.P., Gaeta M.L., de Araujo Mariath J.E., Margis R., Margis-Pinheiro M. (2013). The Mitochondrial Glutathione Peroxidase GPX3 Is Essential for H_2_O_2_ Homeostasis and Root and Shoot Development in Rice. Plant Sci..

[83] Riyazuddin R., Bela K., Horváth E., Rigó G., Gallé Á., Szabados L., Fehér A., Csiszár J. (2019). Overexpression of the Arabidopsis Glutathione Peroxidase-like 5 Gene (AtGPXL5) Resulted in Altered Plant Development and Redox Status. Environ. Exp. Bot..

[84] Riyazuddin R., Bela K., Poór P., Szepesi Á., Horváth E., Rigó G., Szabados L., Fehér A., Csiszár J. (2022). Crosstalk between the Arabidopsis Glutathione Peroxidase-Like 5 Isoenzyme (AtGPXL5) and Ethylene. Int. J. Mol. Sci..

[85] Milla M.A.R., Maurer A., Huete A.R., Gustafson J.P. (2003). Glutathione Peroxidase Genes in Arabidopsis Are Ubiquitous and Regulated by Abiotic Stresses through Diverse Signaling Pathways. Plant J..

[86] Herbette S., de Labrouhe D.T., Drevet J.R., Roeckel-Drevet P. (2011). Transgenic tomatoes showing higher glutathione peroxidase antioxidant activity are more resistant to an abiotic stress but more susceptible to biotic stresses. Plant Sci..

[87] Iqbal A., Yabuta Y., Takeda T., Nakano Y., Shigeoka S. (2006). Hydroperoxide reduction by thioredoxin-specific glutathione peroxidase isoenzymes of Arabidopsis thaliana. FEBS J..

[88] Navrot N., Collin V., Gualberto J., Gelhaye E., Hirasawa M., Rey P., Knaff D.B., Issakidis E., Jacquot J.P., Rouhier N. (2006). Plant glutathione peroxidases are functional peroxiredoxins distributed in several subcellular compartments and regulated during biotic and abiotic stresses. Plant Physiol..

[89] Kalapos B., Juhász C., Balogh E., Kocsy G., Tóbiás I., Gullner G. (2021). Transcriptome profiling of pepper leaves by RNA-Seq during an incompatible and a compatible pepper-tobamovirus interaction. Sci. Rep..

[90] Khan M.I.R., Jahan B., AlAjmi M.F., Rehman M.T., Khan N.A. (2020). Ethephon Mitigates Nickel Stress by Modulating Antioxidant System, Glyoxalase System and Proline Metabolism in Indian Mustard. Physiol. Mol. Biol. Plants.

[91] Tomlinson J.A. (1970). Turnip mosaic virus. MI/AAB Descriptions of Plant Viruses.

[92] Walsh J.A., Jenner C.E. (2002). *Turnip mosaic virus* and the quest for durable resistance. Mol. Plant Pathol..

[93] Jenner C.E., Keane G.J., Jones J.E., Walsh J.A. (1999). Serotypic variation in *Turnip mosaic virus*. Plant Pathol..

[94] Arous S., Harmon C.L., Capobianco H.M., Polston J.E. (2018). Comparison of genus-specific primers in RT-PCR for the broad-spectrum detection of viruses in the genus Potyvirus by plant diagnostic laboratories. J. Virol. Methods.

[95] Kozieł E., Otulak-Kozieł K., Bujarski J.J. (2018). Ultrastructural analysis of *Prune dwarf virus* intercellular transport and pathogenesis. Int. J. Mol. Sci..

[96] Kozieł E., Otulak K., Lockhart B.E.L., Garbaczewska G. (2017). Subcelullar localization of proteins associated with *Prune dwarf virus* replication. Eur. J. Plant Pathol..

[97] Kozieł E., Otulak-Kozieł K., Bujarski J.J. (2020). Modifications in Tissue and Cell Ultrastructure as Elements of Immunity-Like Reaction in *Chenopodium quinoa* against Prune Dwarf Virus (PDV). Cells.

[98] Otulak-Kozieł K., Kozieł E., Lockhart B.E.L., Bujarski J.J. (2020). The Expression of Potato Expansin A3 (*StEXPA3*) and Extensin4 (*StEXT4*) Genes with Distribution of StEXPAs and HRGPs-Extensin Changes as an Effect of Cell Wall Rebuilding in Two Types of PVY^NTN^–*Solanum tuberosum* Interactions. Viruses.

[99] Otulak-Kozieł K., Kozieł E., Lockhart B.E.L. (2018). Plant cell wall dynamics in compatible and incompatible potato response to infection caused by *Potato virus Y* (PVYNTN). Int. J. Mol. Sci..

[100] Luschin-Ebengreuth N., Zechmann B. (2016). Compartment-specific investigations of antioxidants and hydrogen peroxide in leaves of *Arabidopsis thaliana* during dark-induced senescence. Acta Physiol. Plant..

[101] Kranner I., Varma A. (2012). Determination of glutathione, glutathione disulphide and two related enzymes, glutathione reductase and glucose-6-phosphate dehydrogenase, in fungal and plant cells. Mycorrhiza Manual.

[102] Borniego M.L., Molina M.C., Guiamét J.J., Martinez D.E. (2020). Physiological and Proteomic Changes in the Apoplast Accompany Leaf Senescence in *Arabidopsis*. Front Plant Sci..

[103] Vanacker H., Carver T.L.M., Foyer C.H. (1998). Pathogen induced changes in the antioxidant status of the apoplast in barley leaves. Plant Phyiol..

[104] Vanacker H., Foyer C.H., Carver T.L.M. (1998). Changes in apoplastic antioxidants induced by powdery mildew attack in oat genotypes with race non-specific resistance. Planta.

[105] Weimar M., Rothe G. (1987). Preparation of extracts from mature spruce needles for enzymatic analysis. Physiol. Plant..

[106] Gullner G., Komives T., Király L., Schröder P. (2018). Glutathione S-transferase enzymes in plant-pathogen interactions. Front. Plant Sci..

[107] TAIR Database. https://www.arabidopsis.org/.

[108] Islam S., Das Sajib S., Sultana Jui Z., Arabia S., Islam T., Ghosh A. (2019). Genome-Wide identification of glutathione S-transferase gene family in pepper, its classification, and expression profiling under different anatomical and environmental conditions. Sci. Rep..

[109] Islam S., Rahman I.A., Islam T., Ghosh A. (2017). Genome-wide identification and expression analysis of GST gene family in tomato: Gaining an insight to their physiological and stress-specific roles. PLoS ONE.

[110] Bela K., Riyazuddin R., Horváth E., Hurton Á., Gallé Á., Takács Z., Zsigmond L., Szabados L., Tari I., Csiszár J. (2018). Comprehensive analysis of antioxidant mechanisms in Arabidopsis glutathione peroxidase-likemutants under salt-and osmotic stress reveals organ-specific significance of the AtGPXL’s activities. Environ. Exp. Bot..

[111] Tate S.S., Meister A. (1985). *γ*-Glutamyl transpeptidase from kidney. Meth. Enzymol..

[112] Burian M., Podgórska A., Ostaszewska-Bugajska M., Szal B. (2022). Respiratory Burst Oxidase Homolog D as a Modulating Component of Oxidative Response under Ammonium Toxicity. Antioxidants.

[113] Wu Y., Eghbali M., Ou J., Lu R., Toro L., Stefani E. (2010). Quantitative determination of spatial protein-protein correlations in fluorescence confocal microscopy. Biophys. J..

[114] Manders E.M., Stap J., Aten J.A. (1992). Dynamics of three-dimensional replication patterns during the S-phase, analyzed by double labeling of DNA and confocal microscopy. J. Cell Sci..

