# Supplementary material for: Glutathione Contribution in Interactions between Turnip mosaic virus and Arabidopsis thaliana Mutants Lacking Respiratory Burst Oxidase Homologs D and F
Source: Int J Mol Sci. 2023 Apr 12;24(8):7128. doi: 10.3390/ijms24087128 (PMC10138990; doi:10.3390/ijms24087128)
Supplement: Supplementary file 1 [file ijms-24-07128-s001.zip › ijms-2269369-supplementary.pdf]

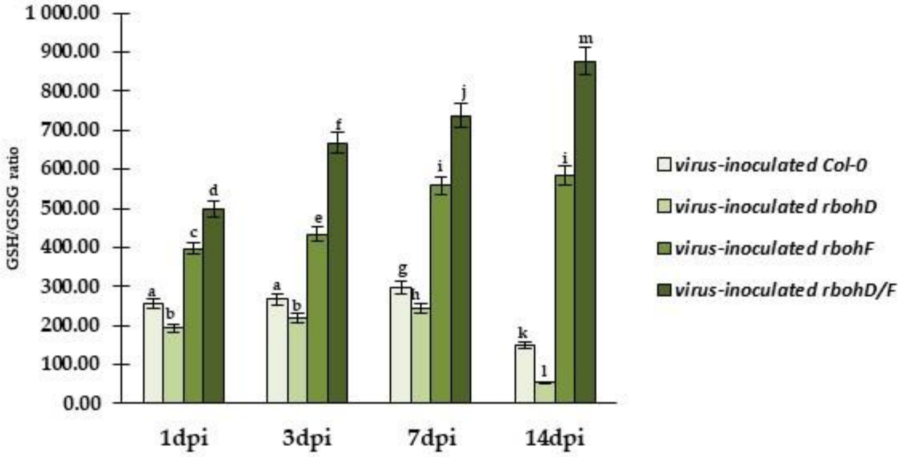

Figure S1. The mean GSH/GSSG ratio in TuMV- and mock-inoculated Col-0, *rbohD, rbohF, and rbohD/F* plant leaves between 1 and 14 dpi. Using ANOVA and Tukey's HSD test, the mean GSH/GSSG ratio were calculated at $p < 0.05$. Statistically significant values are indicated by different letters above the bars.

Table S1. Heatmap of PCC for cellular glutathione (localization and content), host genes expression (*AtGGT1*, *AtGSTU1*, *AtGSTU13*, *AtGSTU24*) and TuMV levels in virus-inoculated Col-0, *rbohD*, *rbohF* and *rbohD/F* plants from 7 to 14 dpi. PCC matrix values are presented pairwise for specific time dpi and marked with colors, from very dark green (PCC = 1) to bright green (PCC = -1).

Levels for TuMV		Localization of total gluthatione in cell		Gluthatione content in cell				Host Gene Expression											
				Levels of GSH		Levels of GSSG		Normalized expression of *AtGGT1*		Normalized expression of *AtGSTU1*		Normalized expression of *AtGSTU13*		Normalized expression of *AtGSTU19*		Normalized expression of *AtGSTU24*					
		7dpi	14dpi	7dpi	14dpi	7dpi	14dpi	7dpi	14dpi	7dpi	14dpi	7dpi	14dpi	7dpi	14dpi	7dpi	14dpi				
Col-0	7dpi																				
	14dpi																				
rbohD	7dpi																				
	14dpi																				
rbohF	7dpi																				
	14dpi																				
rbohD/F	7dpi																				
	14dpi																				

-1
-0.5
0
0.5
1

Table S2. . Heatmap of PCC for **cellular glutathione** related enzymatic activity (GGT,GR, GST and GPXL) and TuMV levels in virus-inoculated Col-0, *rbohD*, *rbohF* and *rbohD/F* plants from 7to 14 dpi. PCC matrix values are presented pairwise for specific time dpi and marked with colors, from very dark green (PCC = 1) to bright green (PCC = -1).

Levels for TuMV		Gluthatione related enzymatic activity in cell							
		Enzymatic Activity of GGT		Enzymatic Activity of GR		Enzymatic Activity of GST		Enzymatic Activity of GPXL	
		7dpi	14dpi	7dpi	14dpi	7dpi	14dpi	7dpi	14dpi
Col-0	7dpi								
	14dpi								
rbohD	7dpi								
	14dpi								
rbohF	7dpi								
	14dpi								
rbohD/F	7dpi								
	14dpi								

-1
-0.5
0
0.5
1

Table S3. Heatmap of PCC for **apoplast total glutathione** localization, apoplast GSH and GSSG content, glutathione related enzymatic activity in apoplast and TuMV levels in virus-inoculated Col-0, *rbohD*, *rbohF* and *rbohD/F* plants from 7to 14 dpi. PCC matrix values are presented pairwise for specific time dpi and marked with colors, from very dark blue (PCC = 1) to bright blue (PCC = -1).

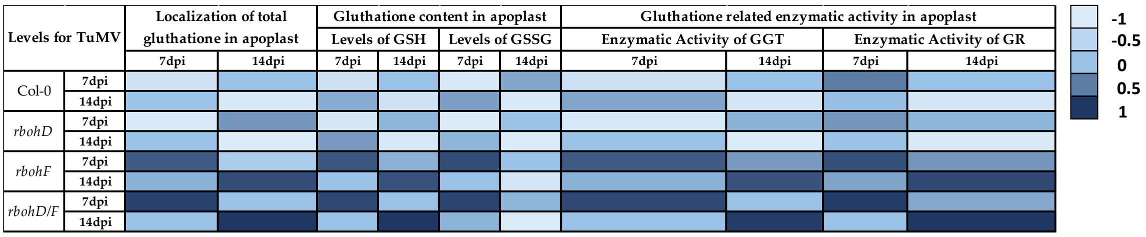

Table S4. Determination of the contamination of the apoplastic extracts (apoplastic washing fluid, AWF) based of total leaf G6PDH activity (in U) and % of activity G6PDH in apoplast. Non -significant cytoplasmatic contamination values are bolded.

G6PDH activity in the total leaf and in the apoplast (%) of tissue from mock and virus inoculated Arabidopsis plants (presented as means ± se)

Combination	Time			
	1dpi	3dpi	7dpi	14 dpi
mock-inoculated Col-0	15.020±0.04	17.000±0.02	15.201±0.03	14.500±0.01
% per cent activity in apoplast	**0.09**	**0.11**	**0.08**	**0.08**
mock-inoculated *rbohD*	17.2310±0.03	18.570±0.01	17.202±0.04	15.290±0.01
% per cent activity in apoplast	**0.08**	**0.075**	**0.071**	**0.07**
mock-inoculated *rbohF*	17.600±0.01	18.890±0.03	17.312±0.01	15.450±0.02
% per cent activity in apoplast	**0.1**	**0.11**	**0.09**	**0.1**
mock-inoculated *rbohD/F*	13.000±0.04	16.022±0.02	14.000±0.04	14.133±0.01
% per cent activity in apoplast	**0.05**	**0.05**	**0.07**	**0.07**
virus-inoculated Col-0	22.400±0.03	25.802±0.04	26.011±0.01	27.000±0.03
% per cent activity in apoplast	**0.2**	**0.2**	**0.23**	**0.23**
virus-inoculated *rbohD*	18.000±0.04	18.520±0.04	21.000±0.03	19.202±0.01
% per cent activity in apoplast	**0.11**	**0.11**	**0.19**	**0.16**
virus-inoculated *rbohF*	23.200±0.04	24.000±0.01	24.32	25.020±0.02
% per cent activity in apoplast	**0.18**	**0.2**	**0.22**	**0.23**
virus-inoculated *rbohD/F*	16.000±0.01	16.500±0.02	17.200±0.04	18.200±0.03
% per cent activity in apoplast	**0.1**	**0.11**	**0.13**	**0.16**

Table S5. Primer sequences for RT-qPCR analyses.

Genes	Forward Primer	Reverse Primer	Concentration in reaction (µM)
		Investigated	
TuMV-CP	5′-CCGGAATTCATGRTITGGTGYATIGAIAAYGG-3′	5′-CGCGGATCCGCIGYYTTCATYTGIRIIWKIGC-3′	0.5
AtGGT1	5′-CGGGGGCATGTGGAATCAGATC-3′	5′-CTCTGTTTGGTATCAGCTGATGGTAG-3	0.5
AtGSTU1	5′-GCAGTGAGGGGATGTATTC-3′	5′-TTTCGTAGGCAAGAAGTATCT-3′	0.5
AtGSTU13	5′-CGCAAAGCAAAAGTTCAATGT-3′	5′-TGGCACAAAACACAGACAAAT-3′	0.5
AtGSTU19	5′-ATGATGCTCAGAGGAAGGTG-3′	5′-ATAGCCAAAGTCATCGCCAC-3′	0,5
AtGSTU24	5′-AAGGTGAGGAGCAAGAAGCA-3′	5′-ACATACCCAAAAGTTTCGTCTC-3′	0.5
		Reference	
AtEF1a	5′-CACCACTGGAGGTTTTGAGG -3′	3′-TGGAGTATTTGGGGGTGGT -5′	0.5
AtF-Box	5′-GCTTGCACACGCCATATCAAT-3′	3′-TGGATTTTACCACCTTCCGCA-5′	0.5

Table S6. Conditions of the RT-qPCR for the reference genes (*).

Program	Parameters
Preliminary denaturation	95 °C for 5 min
Amplification (35 cycles)	95 °C for 10 s<br>58 °C for 10 s<br>72 °C for 20 s *
Melting curve	65–95 °C; 0.1 °C/s

* Fluorescence signal reading was taken at the final stage
